# Partial river flow recovery with forest age is rare in the decades following establishment

**DOI:** 10.1111/gcb.14954

**Published:** 2020-01-19

**Authors:** Laura Bentley, David A. Coomes

**Affiliations:** ^1^ Department of Plant Sciences in the Conservation Research Institute University of Cambridge Cambridge UK

**Keywords:** catchment, forest age, forestation, meta‐analysis, potential evapotranspiration, precipitation, river flow recovery

## Abstract

Forest regeneration and expansion are occurring in many countries, with 80 million ha established from 2000 to 2012 under the Bonn accord and 17.5 million ha established from 1990 to 2005 according to the Food and Agriculture Organisation. Multiple reviews have linked increasing forest cover with reduced river flow and potentially detrimental effects downstream. Previous reviews have investigated trends in river flow response over time, but the influence of forest age remains uncertain. Partial river flow recovery (towards non‐forested conditions) has been reported in decades following forest establishment, but the role of climate in driving these trends has not been explored. Here, we evaluate river flow trends in 43 studies following forest establishment, which provide sufficient information to distinguish the effects of ageing forests from variable climate. Our meta‐analysis supports previous findings showing that forestation reduces annual river flow (by 23% after 5 years and 38% after 25 years) with greater reductions in catchments with higher mean annual precipitation, larger increases in forest cover, and which were idle, rather than agricultural land, prior to forestation. The impact of forests on river flow is sensitive to annual precipitation and potential evapotranspiration, but responses are highly variable. Forests affect river flow less when annual precipitation is low, and sensitivity to precipitation decreases as catchment aridity increases. The majority of catchments demonstrated persistent river flow declines after forest establishment. However, nine catchments showed partial flow recovery after an initial decrease, with peak flow reductions at an average age of 15 and across a range of tree species. The mean rate of recovery was 34 mm/year over 5 years. Partial flow recovery with forest age cannot be commonly expected, however, and forestation programmes should take into account that changes to annual river flow are likely to persist for up to five decades.

## Graphical abstract

Forestation reduces annual river flow in many catchments worldwide. We build on previous systematic reviews to show how forestation affects river flow through time, as a function of forest age and as a result of variable climate. We find there is little evidence at the catchment scale that river flows will partially recover with forest age, and that converting agricultural land to forest has a smaller effect on river flow than converting idle land.

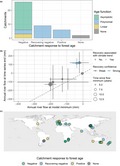

## 1 INTRODUCTION

Over the last 20 years, increasing regional and global forest cover has been promoted for a diverse set of reasons, including erosion control, protection of biodiversity, carbon storage and commercial opportunity (Carle, 2006; Dave et al., 2018; Secretariat of the Convention on Biological Diversity, 2014; Zhou, Zhao, & Zhu, 2012). Concern for biodiversity, resilience and ecosystem functions has increasingly governed forestation and reforestation initiatives, promoting the planting of more diverse stands and native species where appropriate (Brockerhoff, Jactel, Parrotta, Quine, & Sayer, 2008; Chazdon, 2008; Galik & Jackson, 2009; Lamb, 2018). In large areas, Mediterranean and Latin America forests are regenerating naturally on marginal and abandoned lands (Bowen, McAlpine, House, & Smith, 2007; García‐Ruiz & Lana‐Renault, 2011) and further commitments to establish forests within landscape restoration initiatives are yet to be enacted, including India's CAMPA fund (Compensatory Afforestation Fund Management And Planning Authority) and a further 80 million ha pledged under the Bonn accord. In this paper, we will refer to all practices as forestation, which we define as a change in land cover from a stable, non‐forested state to a forested one, independent of the long‐term history of forest cover. In order to achieve the ambitious goals that scientists, national and international policies are championing (Chazdon et al., 2016; Griscom et al., 2017; UNFCCC, 2015), a range of forestation strategies will be necessary, appropriate to the specified objective and the national and local context. However, concerns have been raised about the trade‐offs between forest cover and other environmental services, which might be more abundant in a non‐forested state and on which local communities may depend.

There is widespread agreement that forest establishment is associated with a decrease in annual river flow locally, primarily as a result of increasing transpiration and interception rates (Bosch & Hewlett, 1982; Bruijnzeel, 2004; Filoso, Bezerra, Weiss, & Palmer, 2017; Hamilton, 1992; Jackson et al., 2005; Wei et al., 2018). Significant concern has been voiced about the susceptibility of forested catchments to water shortages as a result of changing hydrology (Dymond, Ausseil, Ekanayake, & Kirschbaum, 2012; Jackson et al., 2005). For many years, changing hydrology as a result of forest cover has been studied through its consequences for river flow (Bosch & Hewlett, 1982; Harrold, Brakensiek, McGuinness, Amerman, & Dreibelbis, 1962). River flow is thought to be equal to the difference between precipitation and evapotranspiration and interception, with changes in ground water storage generally discounted at annual timescales (Bari, Smettem, & Sivapalan, 2005; Zhang, Dawes, & Walker, 2001). Whilst water loss from forests to the atmosphere can generate substantial precipitation downwind (Ellison, Futter, & Bishop, 2012; Ellison et al., 2017; Sheil, 2018 although see Angelini et al., 2011), water vapour is unlikely to be entirely recaptured within the same catchment from which it was released, particularly when the catchments are small (van Dijk & Keenan, 2007). Despite the additional role of forests in stimulating rainfall via the release of volatile organic compounds (Pöhlker et al., 2012), forestation is widely reported to result in a net river flow decline for the same catchment (Evaristo & Mcdonnell, 2019; Jackson et al., 2005; Peel, 2009). The impact of forestation on river flow has been reported to increase with mean annual precipitation (MAP) and forested area (Bosch & Hewlett, 1982; Farley, Jobbágy, & Jackson, 2005; Peel, McMahon, & Finlayson, 2010) and more recently with actual evapotranspiration (Evaristo & Mcdonnell, 2019). Larger river flow responses are reported when afforesting grassland rather than shrubland (Farley et al., 2005) and variation in river flow response by forest type (FT) is frequently reported (Farley et al., 2005; Peel et al., 2010; Zhang et al., 2017). Changes in river flow following afforestation are also thought to be more negative than those of reforestation as a result of the added benefits of increase infiltration from establishment on degraded soils (Bruijnzeel, 2004). Areas that were forested historically may have been through substantial transformations in the intervening period, further influencing responses to the reestablishment of forests (Jackson & Hobbs, 2009). These consequences are drawing attention in the context of regional planning and natural capital assessment (Garcia‐Chevesich, Neary, Scott, & Benyon, 2017; Dymond et al., 2012; Jackson et al., 2005; Jones et al., 2017). It is recognized that whether a decrease in river flow constitutes an ecosystem service or disservice is context specific (Dittrich, Ball, Wreford, Moran, & Spray, 2018; Dymond et al., 2012). However, significant questions remain regarding long‐term trends in river flow response to forestation, and how responses will be affected by an increasingly variable climate (Blöschl et al., 2007; Egginton, Beall, & Buttle, 2014; Locatelli & Vignola, 2009; Yan et al., 2019). Although spontaneous forest regeneration may be key to facilitating large increases in forest cover (Yu et al., 2019), considerably less is known about the hydrological consequences of this process (Peel, 2009), despite important work on this question in South Africa and China (Turpie, Marais, & Blignaut, 2008; Yu et al., 2019). It is important to improve our understanding of the impacts of forestation on water supplies over time, under a range of conditions, and as a result, the potential ecosystem service costs to local regions.

The majority of reviews that compare river flow responses to forestation report average changes in flow per catchment (Evaristo & Mcdonnell, 2019; Filoso et al., 2017; Zhang et al., 2017), and few have examined temporal trends at annual or sub‐annual timescales (Farley et al., 2005; Jackson et al., 2005). Whilst the former benefit from a much larger data pool for spatial analyses, information is lost regarding the progression of river flow responses through time. In addition to mean changes in river flow, understanding: (a) how rapidly changes in river flow occur; (b) the magnitude of variation in the system and (c) what the ultimate stable state of the system might be, is key to interpreting how the benefits and costs of forest cover will interact through time (Asbjornsen et al., 2011; Ellison et al., 2017; Farley et al., 2005; Vertessy, Watson, & O'Sullivan, 2001; Vose et al., 2011). This is particularly true in the context of climate change, which is expected to have significant implications for water security across vast regions (IPCC, 2014). Forest water use is known to respond substantially to annual variation in climate (Llorens et al., 2010; Plaut et al., 2013). In arid regions, forests are more likely to be water limited and responsive to pulses in water availability with increased transpiration, whereas humid catchments are more likely to be energy limited (Asbjornsen et al., 2011). Where water is abundant, changes in interception and evaporative demand are responsible for the majority of increased evapotranspiration following forest establishment (Bruijnzeel, 1990). Climate change is expected to result in greater climatic variation around the world and understanding how these changes translate to the catchment scale is important, but it is also important to account for these processes if we are to get a reliable impression of the effects of forest age on catchment hydrology. No prior review of river flow responses through time has separated the consequences of changing climate from those of forest age.

Understanding how catchment hydrology is likely to change in the decades following forestation will be crucial for the sustainability of forestation initiatives. Evidence for reduced forest transpiration with age can be found in maturing pine (Delzon & Loustau, 2005; Mencuccini & Grace, 1996) and eucalypt stands (Haydon, Benyon, & Lewis, 1997; Vertessy et al., 2001) where overstorey transpiration declined by approximately 40%–70% over 45 years from a maximum at approximately 10 years of age. These observations, and age‐related declines in net primary productivity, have been linked to reduced leaf area index, reduced sap wood area index and reduced transpiration per unit leaf area with age (Haydon et al., 1997; Ryan, Binkley, & Fownes, 1997; Vertessy et al., 2001). Interception rates have also been reported to decrease with age as a result of spatial clumping, and reduced leaf area index (Barbier, Balandier, & Gosselin, 2009; Vertessy et al., 2001), although this is highly dependent on species‐specific growth forms (Huber & Iroumé, 2001). These observations are associated with even aged, monospecific stands and it is likely that successional turnover would offset many of these patterns at the catchment scale. However, greater rates of infiltration at intermediate stand densities may also lead to an increase in river flow as naturally regenerating forests develop (Ilstedt et al., 2016). Where forests establish on highly degraded soils, increasing infiltration can significantly influence catchment hydrology (Bruijnzeel, 2004; Wilcox & Huang, 2010). As we use non‐forested land cover as a baseline in this study, we would not expect river flow to ultimately return to baseline levels, but partial recovery may be observed. If partial river flow recovery (hence forth, river flow recovery) following forestation is widespread, it will have substantial implications for the long‐term trade‐offs between carbon storage and water security in newly forested regions. However, uncertainty remains about the generality of prior observations and whether catchment level observations are driven by forest ageing processes. At the catchment scale, partial river flow recovery has been reported two decades after afforestation with pine species, in a global systematic review using a polynomial relationship, with no clear evidence of recovery in eucalypt plantations, attributed to shorter rotation lengths (Farley et al., 2005). Multiple catchments previously analysed in the context of flow recovery were subject to partial deforestation or destruction during the studied time series (Farley et al., 2005; Scott, Prinsloo, Moses, Mehlomakulu, & Simmers, 2000), which is known to lead to long‐lasting increases in river flow (Bosch & Hewlett, 1982) and must be excluded to determine that signals of river flow recovery are due to forest age. Despite the valuable insights provided by Farley et al. (2005) and Jackson et al. (2005) as the first systematic reviews to examine river flow responses to forestation through time, the influence of temporal variation climate was not accounted for, despite widespread directional trends in climate reported over recent decades. We believe that accounting for the role of temporal variation in precipitation and evaporative demand, and separating it from that of forest age, is an important step to understanding the long‐term ramifications of forestation on river flow. As such, further investigation is required to establish whether river flow recovery following forestation is widespread, and the magnitudes of river flow recovery that can be expected as forests age.

In this paper, we systematically review the factors driving river flow responses to forestation through time. For the first time, we explicitly disentangle the effects of forest age from temporal variation in climate, for multiple sites spanning a range of climatic conditions and land use histories. We separate temporal variation in climate at the catchment scale (referred to as temporal or annual variation) from variation in mean catchment climates (referred to as spatial or between catchment variation). We ask: (a) How is the effect of forestation on river flow affected by variable climate and land use history? and (b) What is the long‐term trajectory of river flow in the decades following forest establishment? We hypothesize that increases in annual precipitation will result in larger decreases in annual river flow following forest establishment, as a result of greater rates of transpiration and interception. We expect this effect to vary with catchment aridity, and to be largest in water‐limited catchments where increased transpiration will likely be observed, in addition to increased interception, which will be observed universally. We expect greater annual potential evapotranspiration (PET) to drive larger decreases in river flow after forestation, as a result of greater evaporative demand, and that this effect will be strongest in humid catchments where interception represents a larger proportion of evapotranspiration and forests are less likely to be water limited. We hypothesize that between‐catchment variation in the rate of change in river flow as forests age will be significantly affected by MAP, the percentage of the catchment forested, whether a catchment was historically forested and whether newly forested land was previously used for agriculture. We hypothesize that larger decreases in river flow will be associated with catchments where a larger area is converted to forest, where MAP is larger, where there is no reported history of forest cover and where no agricultural land use was reported. We expect increases in forest cover and MAP to interact, resulting in greater decreases in river flow, as water availability and landscape capacity for evapotranspiration will increase simultaneously. Finally, we expect that the effect of forest age on river flow will be negative, resulting in progressively decreasing river flow until forests reach maturity. We also expect that patterns of partial river flow recovery will be smaller and less frequent than previously reported, after accounting for temporal variation in precipitation and confounding forest management.

## 2 MATERIALS AND METHODS

### 2.1 Database

Our literature search identified 567 unique data sources in the form of peer‐reviewed journal articles, conference proceedings and book chapters. Of these, 43 unique catchment experiments fitted our selection criteria, including 14 of the 26 studies reported by Farley et al. (2005). The final data set contains 770 data points, sourced from 13 countries. These studies were selected to satisfy strict inclusion criteria, designed to avoid variables that could confound the effects of forestation, discussed below. All studies report the effect of increasing forest cover on non‐forested land; however, some catchments are reported to have had forest cover historically (16 catchments), whilst others claim to have had no prior forest cover (12 catchments), or do not report whether forest cover existed historically in the catchment (15 catchments). We will refer to all cases as forestation, as previously defined. Forests were actively planted in 38 catchments, established unassisted in three catchments and by a combination of both processes in two catchments. Additional catchment information is provided in Supporting Information S1.

To generate this database, a systematic search of the literature was conducted via Web of Science (1900 to 4 January 2018) to identify catchment studies that investigate the impact of forestation on river flow over time. Relevance screening was carried out on titles and abstracts, followed by the main text. Papers that could not be accessed online were requested from the British Library or Cambridge University Library. Selected studies measured the impact of forestation on annual river flow by comparing river flow in forested areas with a control data set of river flow, given pre‐forestation land cover. Accepted experimental designs were single catchment experiments (21) and paired catchment experiments (22). In single catchment experiments, control river flow data (*Q_Ci_*) are predicted from pre‐forestation river flow (under stable land cover) calibrated against coinciding precipitation data, to account for changing climate. In paired catchment experiments, *Q_Ci_* is the flow of a hydrologically similar catchment (with stable non‐forested land cover), following a calibration between the pre‐forestation flows of the control and treatment catchments, to account for any hydrological dissimilarities, including climate. Data were extracted for quasi‐paired catchments in which no calibration between catchments had been carried out, only if differences in climate between the two catchments could be accounted for. To correct for differences in climate we performed calibrations as described in Table 1 (five catchments), where sufficient data were provided for us to do so. Similarly, if a single catchment experiment did not correct for variable precipitation between control and treatment periods (seven catchments), the study was excluded unless we were able to apply a correction to the control data using the precipitation data provided (Table 1). To ensure that including these catchments did not introduce significant bias to the data set, sensitivity analyses were conducted for the hierarchical linear mixed effects models used and are reported in Supporting Information S9. Corrections on *Q_Ci_* were only performed where a significant calibration regression (type 2 ANCOVA; *p* < .05) with an adjusted *R* ^2^ > .5 was found that satisfied assumptions of normality. Corrections were not made for data points outside the range of the calibration. Any single catchment studies that used a mechanistic catchment model to predict river flow had to report validation analyses for that model for the study to be incorporated.

**Table 1:** A description of control river flow calibration methods, where the data extracted had not been previously calibrated. The least‐squares regressions used predict control flow for values of annual precipitation equal to those in the forested catchment in the *i*th year

Study type	Single catchment, no calibration	Paired catchments, no calibration, no data pre‐forestation	Paired catchments, no calibration, pre‐forestation data is present
Data extracted from primary study	Historical river flow (*Q_HFi_*) compared with river flow following forestation (*Q_Fi_*)	Control catchment river flow (*Q_Ci_*) compared with simultaneous forested catchment river flow (*Q_Fi_*). Annual precipitation in control (*P_Ci_*) and forested catchments (*P_Fi_*) differ in any given year	
Calibration regression options	$Q_{\mathrm{HFi}}=\alpha +\beta P_{\mathrm{HFi}}+\varepsilon$	$Q_{\mathrm{Ci}}=\alpha +\beta P_{\mathrm{Ci}}+\varepsilon$	$Q_{\mathrm{HFi}}=\alpha +\beta Q_{\mathrm{HCi}}+\varepsilon$ $Q_{\mathrm{HFi}}=\alpha +\beta P_{\mathrm{HFi}}+\varepsilon$
Prediction of corrected control flow $\left({\hat{Q}}_{i}\right)$	${\hat{Q}}_{i}=\alpha +\beta P_{\mathrm{Fi}}$	${\hat{Q}}_{i}=\alpha +\beta P_{\mathrm{Fi}}$	${\hat{Q}}_{i}=\alpha +\beta Q_{\mathrm{Ci}}$ ${\hat{Q}}_{i}=\alpha +\beta P_{\mathrm{Fi}}$
Description	The historic relationship between precipitation and river flow under control land cover is used to predict river flow under control land cover, for precipitation values during the post‐forestation period	As paired catchments are hydrologically similar, the relationship between control catchment flow and precipitation is used to predict river flow given control land cover and for the precipitation values experienced in the forested catchment	A calibration between historic paired river flows is assumed to account differences due to precipitation If pre‐forestation land cover is stable, a calibration is carried out correcting historical river for differences in precipitation The calibration regression with the highest adjusted *R* ^2^ is implemented

Note

The calibrations used vary according to the initial study design and available data. Variables: *P_i_*, annual precipitation; *Q_i_*, annual river flow; Measurement type (subscripts): *C*, control; *F*, during forestation; *H*, pre‐forestation; *HC*, pre‐forestation in control catchment; *HF*, pre‐forestation in forested catchment.

Data were extracted from primary research papers identified in our search and from papers reviewed by Farley et al. (2005). Studies were not included if treatments were a combination of deforestation and forestation. In contrast to Farley et al. (2005), if felling was reported at any point in the time series all data points following that year were excluded, to remove confounding effects of forest management (Brown, Western, McMahon, & Zhang, 2013). Catchments within a study were required to be hydrologically independent, and therefore, nested catchment studies were not included. To reduce publication bias within the data set, studies that retrospectively investigated of the cause of a known decrease in river flow were not included. If multiple sources reported experiments in the same catchment, data were extracted from the longest time series.

For each selected experiment, we extracted four categories of data: (a) catchment descriptors such as catchment area (km^2^), MAP (mm) and land cover prior to forestation; (b) treatment descriptors including FT (coniferous, broadleaf or mixed) and year of planting; (c) experiment descriptors including treatment duration (years) and the method used to produce the control data set and (d) the experimental data, consisting of time since first planting, area of forest planted per year, control river flow, treatment river flow, annual precipitation and the hydrological year of study. We required that forest age was known through time, and that the percentage of catchment that was forest was reported. Where the change in forest area was not reported for a given year between 2 years of known forest area, the rate of expansion was assumed constant. A full description of extracted data is available in Supporting Information S2. Data presented graphically were extracted using PlotDigitizer (Huwaldt, 2015).

Annual precipitation and PET (calculated using a variant of the Penman–Monteith method) data were extracted from CRU TS4.3 (Harris, Jones, Osborn, & Lister, 2014) using the location and extent of the experimental catchment where provided (30 catchments). Catchment extents were digitized manually. Where insufficient data were provided to reliably digitize catchment extent, climate data were extracted from a circle of equal area centred on the catchment's location to provide a representative sample of variation (12 catchments), or in the absence of catchment area (one catchment), simply from the catchment's location. Extracted precipitation data were only used where studies did not report it (seven catchments). Prior to extraction, CRU TS4 precipitation data were compared to reported precipitation values from our database, to determine the level of agreement between the two data sources (Supporting Information S7). A catchment aridity index was calculated by dividing MAP by mean annual PET. No *Q_Ci_* corrections were based on extracted values for precipitation. To assess how representative the forests in this study are of forests globally, MAP and mean annual temperatures were extracted from WorldClim2 (Fick & Hijmans, 2017) for the period of 1970–2000 and used to determine Whittaker biomes.

### 2.2 Analysis

#### 2.2.1 Quantifying river flow responses to forestation

The absolute difference between annual river flow after forestation (*Q_Fi_*, mm) and control annual river flow (*Q_Ci_*, mm) was used to quantify river flow response to forestation (*Q_i_*, mm).

(1) $$Q_{i}=Q_{\mathrm{Fi}}-Q_{\mathrm{Ci}}.$$

In some studies, forestation occurred over multiple years. To reflect the forest structure influencing water yields, forest age was computed as an area‐weighted mean. To summarize the data set, the effects of forestation on river flow were averaged across studies for each Whittaker biome and for each 5‐year time step. Each average was composed of only one data point from each catchment, within ±0.5 years from the focal forest age. In all cases, reported measurements of precision are standard deviations unless stated otherwise. All analysis was performed using R v 3.5.2 (R Core Team, 2016).

To isolate the effect of temporal variation in climate, annual precipitation and PET were standardized by subtracting MAP (mm) and mean catchment PET (mm), respectively, producing variables for the temporal variation in precipitation within a catchment (*P_Ti_*, mm) and temporal variation in PET (*PET_Ti_*, mm). Change in forest cover was also standardized by the same procedure to form two variables: mean percent forest cover for each catchment, varying only through space (*FC_S_*) and temporal change in forest cover (*FC_Ti_*), standardized by *FC_S_*.

### 2.3 Catchment analysis

Extensive preliminary testing was carried out for each catchment (*j*) to investigate whether a linear, second‐order polynomial or asymptotic function of forest age (*Age_ij_*) was best suited to explain temporal variation in river flow response (*Q_ij_*), along with annual precipitation (*P_Tij_*), annual PET (*PET_Tij_*), polynomial terms for both climate variables and where appropriate, change in forest cover (*FC_Tij_*). The coefficients of *Age_ij_*, ${Age_{\mathrm{ij}}}^{2}$, *P_Tij_*, ${P_{\mathrm{Tij}}}^{2}$, *PET_Tij_*, ${PET_{\mathrm{Tij}}}^{2}$ and *FC_Tij_* are represented by *a*–g respectively (2, 3). Starting values for coefficients *h* and *k* in asymptotic regressions of *Age_ij_* were estimated following visual inspection of the data (3). Linear, polynomial and asymptotic functions of forest age were the preferred model in 2, 19 and 20 catchments, respectively, with two catchments where age was not retained in model structure. Models were fitted for all catchments using least‐squares regression (linear or non‐linear), with normally distributed residual error (*ε*). Model selection was carried out for each starting model structure (2, 3) using the Akaike information criterion (AIC), and final model forms were compared with AIC to select the overall best model structure. When comparing polynomial and asymptotic model structures, asymptotic models were given preference when the difference in AIC was small (<2), as a result of the simpler mechanistic assumptions associated with the effect of forest age on river flow reaching a stable state, rather than changing direction as a result of the processes previously discussed. AIC values for final model structures compared are provided in Supporting Information S5. Where explanatory variables were found to be correlated (Pearson's correlation coefficient, |*r*| > .7), each variable was tested in the model separately, to avoid coefficient inflation and the preferred final model retained. Climatic variables and age were correlated in eight catchments, and forest cover and age were correlated in 15 catchments. All functions of forest age were fitted through the origin (Zar, 1999), representing the year prior to forestation, where control and forested river flows are necessarily equal, barring measurement error. Insufficient degrees of freedom prevented some model forms being tested for specific catchments.

(2) $$\begin{array}{cc} Q_{\mathrm{ij}} & =0+a\;Age_{\mathrm{ij}}+b\left({Age_{\mathrm{ij}}}^{2}\right)+c\;P_{\mathrm{Tij}}+d\left({P_{\mathrm{Tij}}}^{2}\right)+e\;PET_{\mathrm{Tij}}\\ & \;+f\left({PET_{\mathrm{Tij}}}^{2}\right)+g\;FC_{\mathrm{Tij}}+\varepsilon , \end{array}$$

(3) $$\begin{array}{cc} Q_{\mathrm{ij}} & =0+h\left(1-\exp\left(-\exp\left(k\right)\;Age_{\mathrm{ij}}\right)\right)+c\;P_{\mathrm{Tij}}+d\left({P_{\mathrm{Tij}}}^{2}\right)+e\;PET_{\mathrm{Tij}}\\ & \;+f\left({PET_{\mathrm{Tij}}}^{2}\right)+g\;FC_{\mathrm{Tij}}+\varepsilon . \end{array}$$

#### 2.3.1 Q1: How is the effect of forestation on river flow affected by variable climate and land use history?

In order to quantify trends in river flow response both through time and between catchments simultaneously, we used a hierarchical linear mixed effects model with a Gaussian error distribution, applied to the entire data set. River flow response across all catchments was modelled as a function of temporal explanatory factors (4), with coefficients *a*–*d* fitted at the catchment level (5–8). Temporal variables included forest age (*Age_i_*), ${Age_{i}}^{2}$, within catchment variation in precipitation (*P_Ti_*) and within catchment variation in PET (*PET_Ti_*). Polynomial climate terms were not included due to limitations in explanatory power. A polynomial function of age was used as this was able to approximate all model fits well, given separate *b* coefficients for each catchment (6). Within catchment variation in forest cover (*FC_Ti_*) was not included as it was dropped from all single catchment models. The coefficients of *Age_i_*, ${Age_{i}}^{2}$, *P_Ti_* and *PET_Ti_* are represented by *a*, *b*, *c* and *d* respectively (4). The values of coefficients *a*,* c* and *d* are modelled as a function of spatial explanatory variables (5, 7, 8). Catchment sensitivities to variation in annual precipitation (*c*) and to annual PET (*d*) were modelled as a function of mean catchment aridity, with coefficients *n* and *r* (intercepts) and *o* and *s* (slopes; 7, 8). The sensitivity of a catchment to increasing forest age (*a*) was modelled as a function of MAP, *FC_S_*, the presence or absence of historical forest cover (*HF*, present, absent or unknown) and previous land use (*PLU*, agriculture, idle or other), using coefficients *e*, *f*, *g*, *k* and *l* (5). An interaction term between *MAP* and *FC_S_* was included via coefficient *h*, as we expect the magnitude of the response to *MAP* to increase with the area of forest present. Coefficient *e* is the intercept value of *a* when *MAP* and *FC_S_* are zero, *HR* is absent and *PLU* is agriculture. The strength of the polynomial function of age (*b*) is quantified as a single coefficient *m*. Random effects were fitted at the catchment level for coefficients of forest age ($\sigma_{1}^{2}$), the second‐order polynomial of forest age ($\sigma_{2}^{2}$), annual precipitation ($\sigma_{3}^{2}$) and annual PET ($\sigma_{4}^{2}$) a priori. To account for temporal autocorrelation, an autocorrelation‐moving average correlation structure (corARMA) was included with catchment level grouping and three autoregressive parameters, following visual inspection of the autocorrelation and partial autocorrelation structure within model residuals. Model residuals were inspected to ensure compliance with assumptions of normality. Significance of final model terms was assessed via an ANOVA using marginal sum of squares.

(4) $$Q_{i}=a\;Age_{i}+b\;Age_{i}^{2}+c\;P_{\mathrm{Wi}}+d\;PET_{\mathrm{Wi}}+\varepsilon ,$$

where

(5) $$a∼N\left(e+f\;MAP+g\;FC_{B}+h\left(MAP\times FC\right)+k\;HR+l\;PLU,\;\sigma_{1}^{2}\right),$$

(6) $$b∼N\left(m,\;\sigma_{2}^{2}\right),$$

(7) $$c∼N\left(n+o\;\text{Aridity},\;\sigma_{3}^{2}\right),$$

(8) $$d∼N\left(r+s\;\text{Aridity},\;\sigma_{4}^{2}\right).$$

Initial testing for confounded explanatory variables was done in pairwise combinations, using Fisher's tests and ANOVA tests followed by Holm corrections, and Pearson's correlation coefficient, where appropriate. We tested for associations within our chosen explanatory variables and between those and other variables of interest common in the literature. These tests identified a significant correlation between prior land use and prior land cover (*PLC*, grassland, shrubland, other), between *FC_S_* and *FT* (broadleaf, coniferous and mixed) and between *MAP*, mean annual PET and aridity index within our data set. Post hoc tests showed the association between *FC_S_* and *FT* was due to a lower mean forest cover in mixed and unknown forest catchments relative to broadleaf and coniferous forests. Replicate analyses were carried out using alternate model structures for *a*, in which *FT* is incorporated in place of *FC_S_*, *PLC* is incorporated in place of *PLU* and mean annual PET or aridity index in place of *MAP* respectively. All alternate model structures were associated with higher AIC scores than that reported in the main text. Variable association test results and alternate model results are provided in Supporting Information S3. Pearson's correlation tests were used to determine whether catchment climatic variation (both range and standard deviation, in both precipitation and PET) was correlated with mean catchment to ensure that larger climatic variation is not driving larger coefficient estimates for *o* and *s* (7, 8).

#### 2.3.2 Q2: What is the long‐term trajectory of river flow in the decades following forest establishment?

The occurrence of river flow recovery is indicated by the preference of a polynomial function of forest age, with a negative *a* coefficient and a positive *b* coefficient. Model fitting was carried out at the individual catchment level as previously described (2, 3), to quantify the abundance of recovery signals and maximize the model fits for the relevant catchments.

(9) $$\frac{dQ_{\mathrm{ij}}}{{\text{dAge}}_{\mathrm{ij}}}=a+2b\;Age_{\mathrm{ij}}=0,$$

To estimate confidence in river flow recovery signals, model estimates of river flow response were compared between forest ages at the minimum flow value, where Equation 9 is true, *a* is negative and *b* is positive (coefficients are as estimated by Equation 2) and estimates at the final forest age in the time series. If the 95% confidence interval for these two estimates does not overlap, there is strong evidence for recovery in that catchment. This comparison was only made if the predicted minimum occurred within the data set. Pearson's correlation coefficient (*r* > |.7|) was used to identify catchments in which precipitation or PET was confounded with forest age for the recovery phase of the time series specifically. To make rates of recovery across catchments comparable, estimates of river flow response at the minimum flow value were also compared with those 5 years after the minimum, to standardize for the effect of time. We classified river flow responses to forest age in the preferred model of each catchment as negative (*a* in Equation 2 or *h* in Equation 3 is negative), recovering negative (Equation 2 *a* is negative, *b* is positive and the minimum is not after the data set), positive (*a* in Equation 2 or *h* in Equation 3 is positive) or none (no age term retained) in order to represent the variety of responses in the data set. Fisher's exact test was used to determine whether catchment response to forest age was affected by forest age structure (uniform or non‐uniform) or species richness (monospecific or polyspecific) and a Kruskal–Wallis test was used to identify any effect of time series duration on catchment response to forest age. As the objective of this analysis is to determine the long‐term trajectory of catchment river flow following forestation, and whether river flows partially recover with age, this analysis was repeated excluding all data points for which annual flow in the forested catchment was equal to zero. Whilst *Q_Fi_* equals zero, variation in *Q_i_* reflects changes in *Q_Ci_* only, which may cause false signals of river flow decline or recovery. This was the case for 22 data points across five catchments, occurring in the final 4, 5 and 9 years of three time series, and as isolated years within the time series of two catchments. We expect that most variation in *Q_Ci_* is due to variation in annual precipitation or PET and will be accounted for in both analyses. Examples of *Q_Ci_*, *Q_Fi_* and *Q_i_* are provided in Supporting Information S6 where the removal of data was necessary, along with estimated rates of river flow recovery prior to the exclusion of the relevant data points.

## 3 RESULTS

### 3.1 Overall effects of forestation on annual river flow

**Figure 1:**
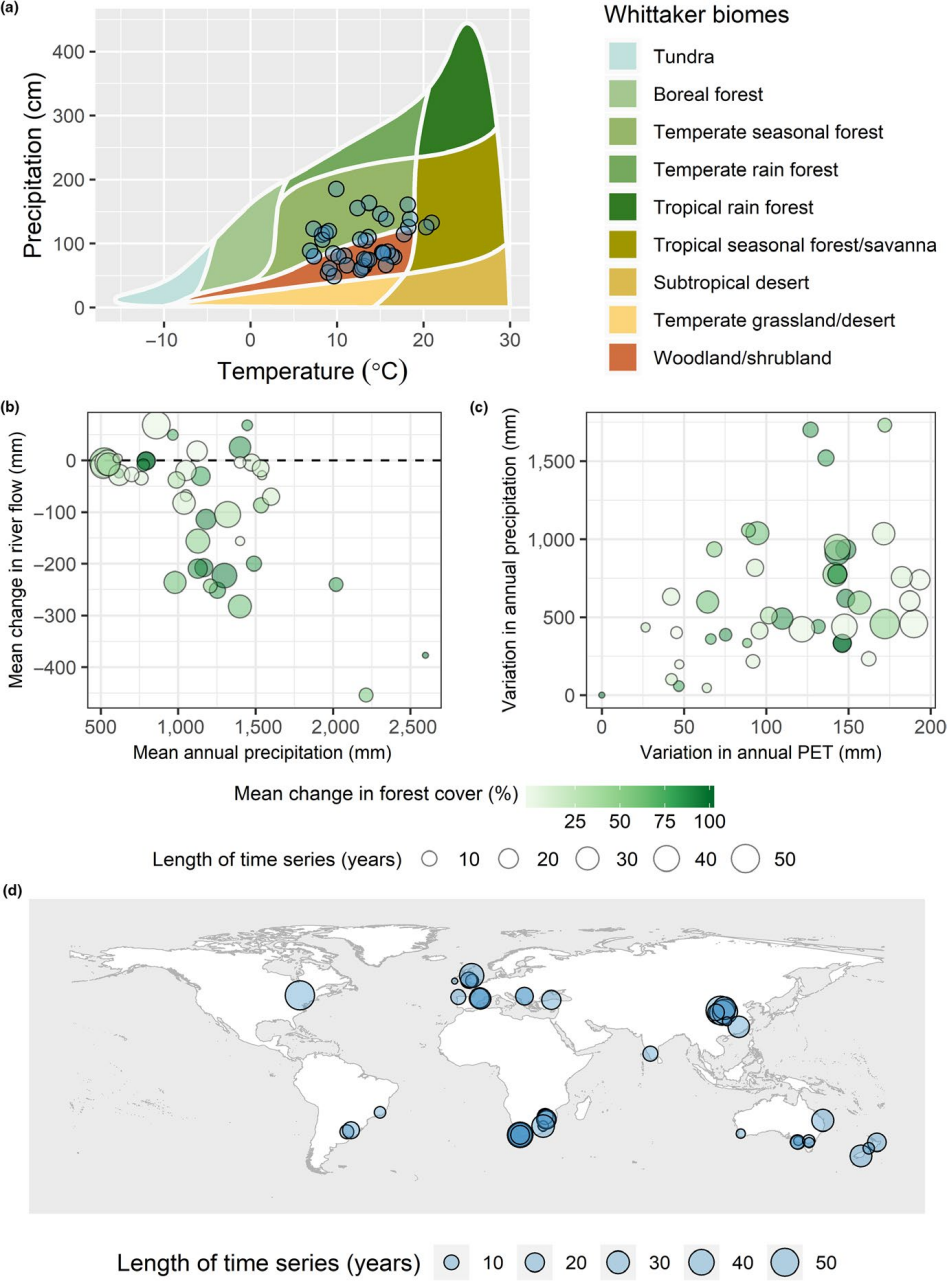
(a) the distribution of catchments across Whitaker biomes, created using the plotbiomes R package (Valentin, 2018); (b) the distribution of mean changes in catchment river flow by mean annual precipitation and mean change in forest cover; (c) the range for reported annual precipitation and annual potential evapotranspiration (*PET*) across catchment time series; (d) the geographic distribution of catchments

The average effect of forestation on river flow varies from −84 mm (−22.5%) at a forest age of 5, to −211 mm (−38.4%) at a forest age of 25. However, in some catchments, decreases of 100% and over 500 mm were reported. Catchments are distributed across three Whittaker biomes and 13 countries (Figure 1). Contrary to classical studies, but in agreement with reviews in recent years, we found dramatic decreases in river flow within the years immediately following forestation. Enormous variation in river flow response to forestation is present between studies, and through time (Table 2). Temperate catchment responses varied through time to a greater degree than those in woodland/shrubland catchments. Tropical forests were underrepresented in this database, with two catchments of tropical seasonal forest present. This literature bias has been widely reported elsewhere. Within the data set, study durations ranged from 2 to 57 years with a mean of 19. Catchment MAP ranged from 517 to 2,597 mm with a mean of 1,157 mm and mean PET ranged from 463 to 1547 mm with a mean of 1,033 mm.

**Table 2:** Summary of the change in river flow after forestation across all bio‐geographic realms

Age	Change in river flow											
	All			Temperate seasonal forest			Tropical seasonal forest/savanna			Woodland/shrubland		
	mm	*SD*	*N*	mm	*SD*	*N*	mm	*SD*	*N*	mm	*SD*	*N*
5	−83.5	124.5	28	−75.0	116.9	13	−197.7	—	1	−103.1	132.0	14
10	−77.6	154.2	26	−49.5	197.9	11	−47.8	—	1	−92.3	119.1	15
15	−106.5	155.7	21	−138.4	232.6	8	—	—	0	−91.5	85.2	13
20	−187.3	183.5	12	−292.1	241.1	3	—	—	0	−151.9	162.6	9
25	−211.4	151.6	9	−274.7	127.6	3	—	—	0	−179.8	163.3	6
30	−130.8	144.2	7	−128.9	190.9	3	—	—	0	−132.3	131.6	4
35	−99.1	136.0	6	15.18	23.0	2	—	—	0	−156.3	132.6	4
40	−54.2	163.3	4	38.9	55.3	2	—	—	0	−147.3	205.6	2

Note

Change in river flow is represented as a change from control river flow (mm). *N* is sample size and *SD* is standard deviation. Sampled data included only one data point per catchment per age class, from within ±0.5 years of the focal age.

#### 3.1.1 Q1: How is the effect of forestation on river flow affected by variable climate and land use history?

**Figure 2:**
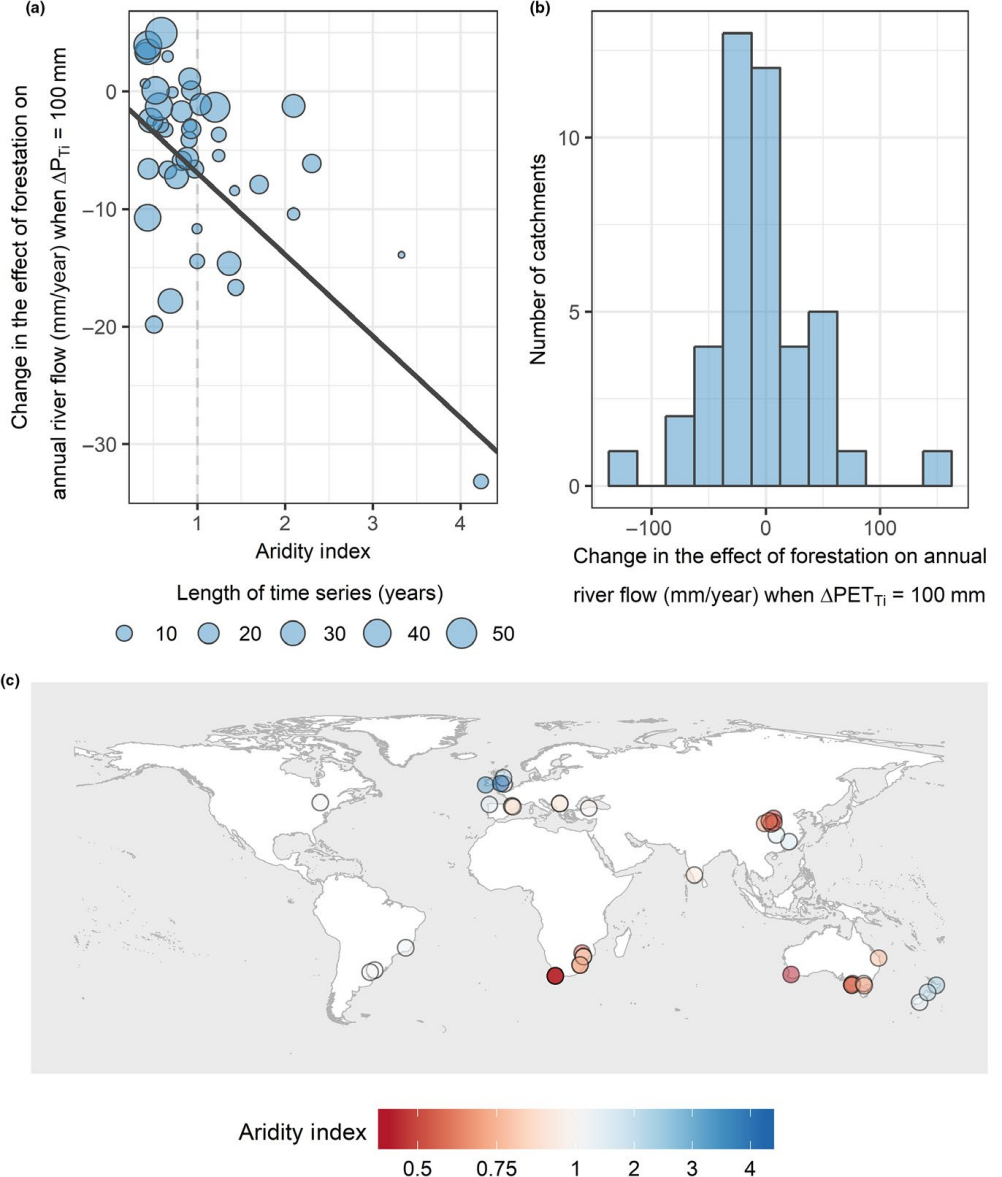
(a) Mixed effect model predictions for the change in the effect of forestation on annual river flow (*Q_i_*) when annual precipitation (*P_Ti_*) increases by 100 mm, as a function of aridity index for each catchment (points) and overall (solid line); (b) mixed effect model predictions of the change in the effect forestation on annual river flow (*Q_i_*) when annual potential evapotranspiration (*PET_Ti_*) increases by 100 mm; (c) the distribution of catchments and associated aridity indices

**Figure 3:**
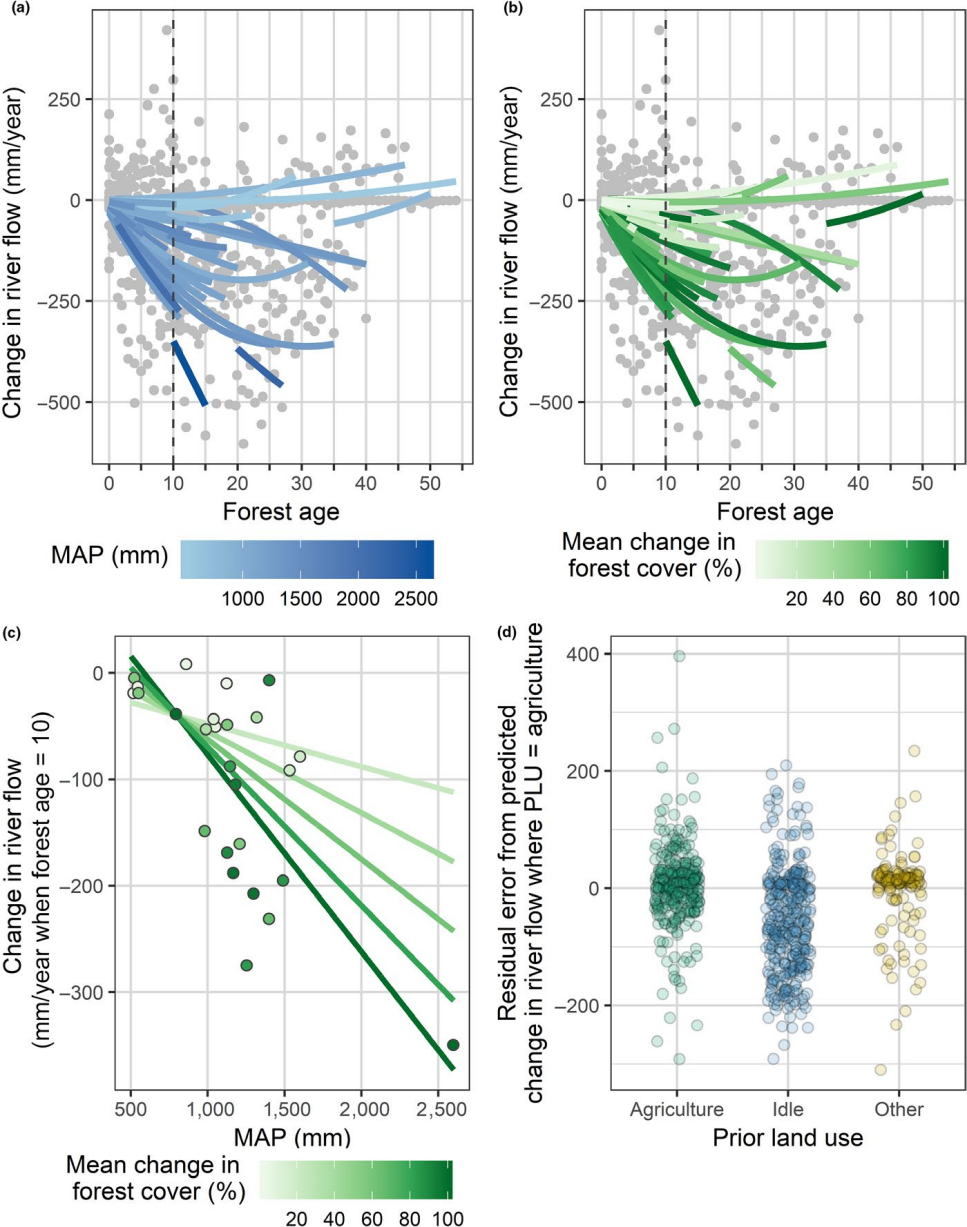
(a–c) Mixed effect model predictions for the change in river flow (mm) when annual rainfall is equal to mean annual precipitation (*MAP*) and annual potential evapotranspiration (*PET*) is equal to mean *PET*, illustrating: (a) catchment *MAP* overlaying raw data; (b) mean change in forest cover as a percent of catchment area (*FC_S_*) overlaying raw data; (c) change in river flow for catchments where forest age is 10 as function of *MAP* and *FC_S_* for catchments reporting data at age 10 (points), and mean predictions, assuming the prior land use (*PLU*) was agriculture (solid lines); (d) residual error between the raw data and predicted change in river flow assuming PLU was agriculture for all points

Temporal variation in river flow response was best described by a positive second‐order polynomial function of forest age, annual precipitation (*P_Ti_*) and annual PET (*PET_Ti_)*. Greater annual precipitation is associated with greater decreases in annual river flow following forestation, suggesting that forest water use and interception increase in response to greater water availability at annual timescales, in accordance with our hypotheses and previous analyses. The sensitivity of catchments to annual precipitation is affected by the aridity of the catchment. Catchments with a larger aridity index (greater MAP and smaller mean PET) respond more strongly to variable annual precipitation (Figure 2a), resulting in a greater reduction in annual river flow when precipitation is high (*n* = 0.015, *F* = 0.22, *p* = .64; *o* = −0.07, *F* = 5.746, *df* = 1, *p* = .0168; 13). However, the small number of catchments with high aridity index in this data set suggests that more studies from humid climates are desirable to confirm this result. The significance of aridity index as an explanatory factor for *c* was found to be dependent on the inclusion of quasi‐paired studies in our analysis, which included the least arid catchments in our data set. Catchment responses to variation in annual PET were not correlated with aridity; however, values for *d* vary substantially between catchments (Figure 2b; *r* = −.13, *F* = 0.18, *df* = 1, *p* = .68; 14). The mean range in annual precipitation and annual PET values for catchments were 630 and 114 mm respectively (Figure 1c). Variation in catchment climate was not correlated with mean climate in any combination of variables tested (Supporting Information S3). Catchment response to forest age is the result of an interaction between MAP and mean change in forest cover (*e* = −5.3, *F* = 2.2, *df* = 1, *p* = .14; *f = *−0.00036, *F* = 0.014, *df* = 1, *p* = .91; *g* = 0.13, *F* = 6.1, *df* = 1, *p* = .01; *h* = −0.00015, *F* = 11, *df* = 1, *p* < .001) and prior land use (*l*_Agriculture_ = −5.3, *l*_Idle_ = −8.1, *l*_Other_ = −4.5, *F* = 3.9, *df* = 2, *p* = .021; 11). Catchments with a higher *MAP* and *FC_S_* are associated with faster rates of river flow decline as forests age (Figure 3). Catchments which were reported to be used for agriculture prior to forestation showed a smaller river flow response to forestation than catchments that were idle. The secondary polynomial function of forest age was positive and significant (*m* = 0.18, *F* = 11.7, *df* = 1, *p* < .001). Coefficient estimates for each catchment are provided in Supporting Information S4. The mean values for *a*, *b*, *c* and *d* are −10.1 (±9.03), 0.176 (±0.154), −0.0561 (±0.0745) and −0.0519 (±0.441) respectively. A high degree of correlation was present between certain random effects (Table 3). Final model AIC was 8,760.4. Variation explained by random effects is shown in Equations (10), (11), (12), (13).

(10) $$Q_{i}=a\;Age_{i}+b\;{Age_{i}}^{2}+c\;P_{\mathrm{Ti}}+d\;PET_{\mathrm{Ti}}+\varepsilon ,$$

where

(11) $$a∼N\left(e+f\;MAP+g\;FC_{S}+h\left(MAP\times FC\right)+l\;PLU,\;\sigma_{1}^{2}=56.4\right),$$

(12) $$b∼N\left(m,\;\sigma_{2}^{2}=0.0353\right),$$

(13) $$c∼N\left(n+o\;\text{Aridity},\;\sigma_{3}^{2}=0.00602\right),$$

(14) $$d∼N\left(r,\;\sigma_{3}^{2}=0.333\right).$$

**Table 3:** Correlation matrix between random effects

	$\sigma_{2}^{2}$	$\sigma_{3}^{2}$	$\sigma_{4}^{2}$
$\sigma_{1}^{2}$	−0.990	0.910	−0.597
$\sigma_{2}^{2}$		−0.958	0.480
$\sigma_{3}^{2}$			−0.212

#### 3.1.2 Q2: What is the long‐term trajectory of river flow in the decades following forest establishment?

**Figure 4:**
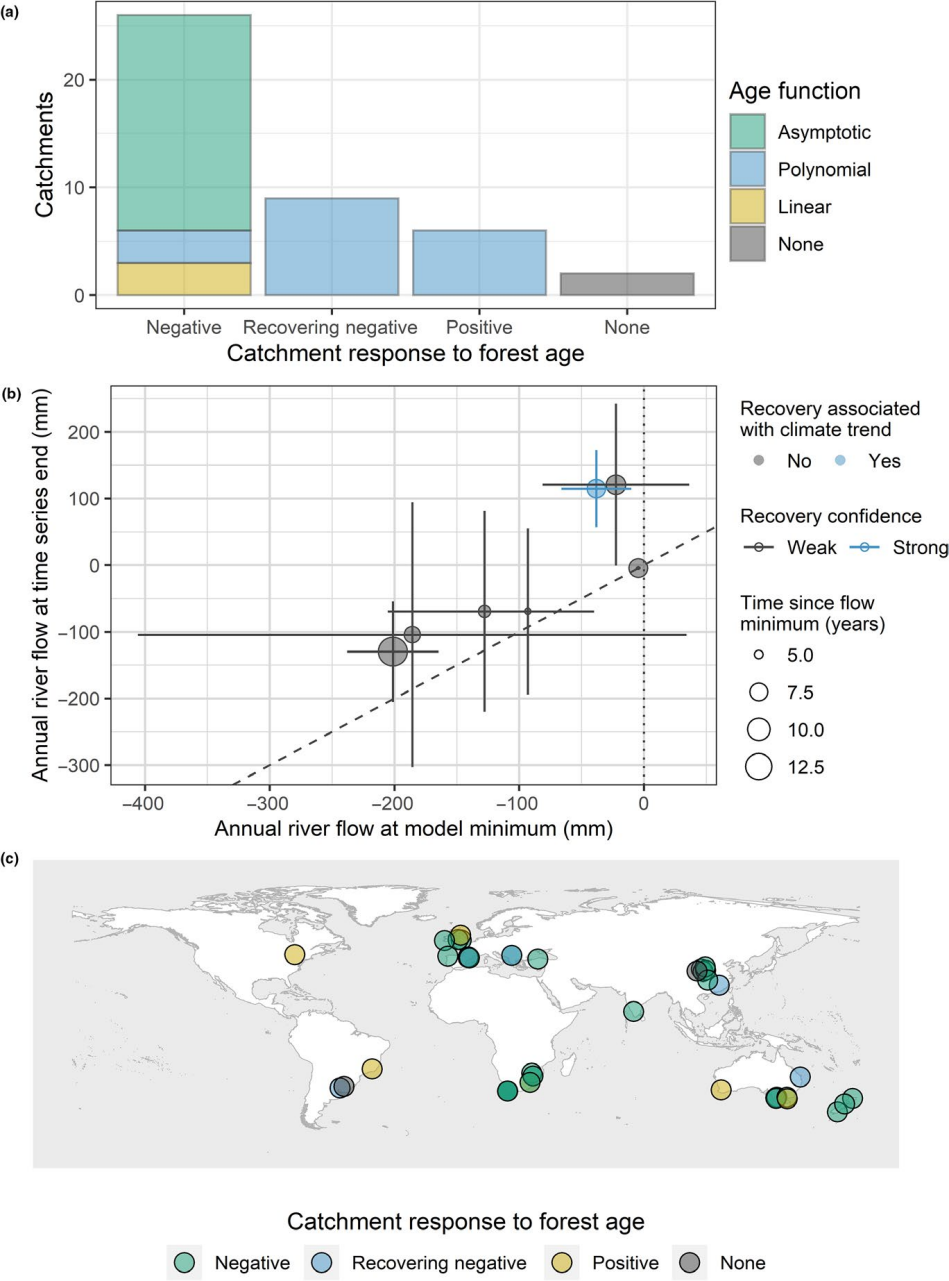
(a) A summary of the relationships observed between change in river flow and forest age; (b) the magnitude of river flow recovery predicted by recovering negative catchment models, from the point at which the effect size is largest to the end of the time series, accompanied by 95% confidence intervals to each estimate. Data points where forested flow (*Q_Fi_*) equalled zero have been removed. The dashed line shows a 1:1 relationship; (c) distribution of catchments and their responses to forest age

Of the 43 catchments, 35 showed an initial decrease in river flow in response to forest establishment, two catchments showed no significant effect of forest age (Luoyugou and Manuel Diaz) and six catchments showed a positive trend with forest age. Of the 35 catchments with a negative initial trajectory, 11 catchments were best described by a second‐order polynomial function of forest age, nine of which have a positive *b* coefficient and seven of which contain the model minimum within the reported time series. Negative linear functions of age were preferred in three catchments and asymptotic fits were preferred in 20 catchments. All catchments showing an initial increase in river flow were best described by a negative polynomial of forest age (Figure 4a). No significant effects of species richness (*p* = .105), age structure (*p* = .452) or time series duration (*Χ* ^2^ = 3.30, *df* = 3, *p* = .348) were found on the response of river flow to forest age. A summary of terms included in each model is provided (Supporting Information S5). Of the catchments with a signal of partial recovery, two were planted with monospecific *Eucalyptus globulus*, one was monospecific *Pinus radiata*, three catchments contained a mixture of *Pinus* and *Quercus* species and one catchment was a mixture of *Robina pseudoacacia*, *Prunus armeniaca* and *Populus davidiana*. Two catchments did not report the species present.

The mean age at which a minimum in river flow response occurs (the largest difference from control flow in partially recovering catchments) is 15.3, with a range of 3–47. The average adjusted *R* ^2^ of recovering negative models was 0.62 (±0.29). Our confidence in the signal of river flow recovery was measured by whether the 95% confidence intervals of model estimates at the minimum flow value and final flow value overlapped. One of 43 catchments reported non‐overlapping confidence intervals (Flamisell catchment; Figure 4b). However, patterns of river flow recovery in which final flow is above that of the control at the end of the time series are unexpected (Flamisell and Escalo catchments). Flamisell catchment's recovery phase is associated with persistent precipitation declines, correlated with forest age (*r* = −.77) although annual precipitation was not found to be significant predictor of river flow response for the time series. Across the seven catchments considered, 5 years after the peak reduction in river flow response the difference between control and forested catchment flow had shrunk by an average of 34.7 (±24.1) mm/year, equivalent to a reduction in the magnitude of river flow responses by 71.3% (±92.7) relative to peak response. The geographic distribution of relevant catchments is shown (Figure 4c).

## 4 DISCUSSION

In the context of carbon storage goals and concerns for water security, the need to anticipate consequences of widespread forest establishment is increasing. We find that negative effects of forestation on catchment river flow are widespread at annual timescales, and are of similar magnitude to those previously reported (Bosch & Hewlett, 1982; Filoso et al., 2017; Jackson et al., 2005). Whilst the average reduction in river flow is approximately one‐third of control river flow, enormous variation is present through both time and space, which we identify as a result of interactions between forest cover, land use history and prevailing climate. This data set is the largest collection of temporal data on river flow responses to forestation of which we are aware. Using it we report new estimates of the interaction between forest age, expansion and climate, and shown the importance of PLUs for expected rates of river flow decline. We also provide the first review of the progression of river flow responses to forest cover as a result of forest age.

### 4.1 Rate of change in river flow response

The effects of agricultural practices, arable and pastoral, are known to influence soil properties by compression, decreasing infiltration and potentially the loss of topsoil and organic matter (Drewry, 2006; Sirimarco, Barral, Villarino, & Laterra, 2018; Wu & Tiessen, 2002). Whilst the magnitude of these changes is a result of the intensity of agricultural practises and their duration, we show that land use history immediately prior to forest establishment is a significant predictor of the rate of river flow response to forest establishment, with catchments that were previously idle showing faster rates of decline than those that were reported as having been used for agriculture. Previous studies have shown that forest establishment on degraded land can substantially increase infiltration and ground water recharge rates (Bruijnzeel, 2004; Ilstedt, Malmer, Verbeeten, & Murdiyarso, 2007; Sillon, Richard, & Cousin, 2003). Although we suspect this to be the driving mechanism behind observed differences, authors rarely reported the details of any changes in soil traits, prior land use intensity or provided more than the land use immediately prior to forest establishment. We also find that whether a catchment is known to have a history of forest cover is a relatively poor indicator of the rate of river flow decline following forest establishment, likely due to the variable time frames between historic forest loss and current forestation, with variable treatment of the catchment in the interim (Jackson & Hobbs, 2009). We believe assessments of infiltration rates and bulk density prior to forest establishment would significantly assist the interpretation of any known land use history. A study of changing infiltration with variable tree cover was carried out by Ilstedt et al. (2016) and across stand ages by Zhang et al. (2019); however, we know of no review examining changes in infiltration rates as forests age. Despite associations between prior land use and land cover within our data set, no significant effect of PLC (grassland, shrubland or other) was found in our alternate model tests, in contrast to what has been reported elsewhere (Farley et al., 2005).

We show a significant relationship between the observed rates of river flow response to forest establishment and both catchment MAP and forest cover, as has been reported elsewhere. We quantify the interdependence of the effects of MAP and forest cover and their interaction with forest age, showing that the ability of forests to adapt to environmental water availability, to an increasing degree with age, is manifest at the catchment scale. This ability is likely due to a combination of structural differences resulting in greater interception and transpiration rates with water availability, the principle driver of river flow response in arid climates (the majority of our data set; Asbjornsen et al., 2011). The interactions between MAP, forest cover and age can substantially influence expected changes in river flow through time. Using the mean coefficient values of our mixed effect model, we estimate that the forestation of a catchment with a MAP of 1,000 mm would result in an addition reduction of river flow by 25 mm/year when the forest is 5 years old, and of 51 mm/year at 20 years of age, relative to a catchment with a MAP of 500 mm, when 50% of the catchment is forested (previously agriculture). If 100% of the catchment is forested, these values increase to 90 and 177 mm/year (one‐third of the mean catchment mean control flow in our data set) respectively. By comparison, we find that an equivalent increase in annual precipitation of 500 mm will result in a 26 mm/year additional reduction in river flow when the catchment mean aridity index is one, and 93 mm/year when the mean aridity index is three. Our results show that wetter years facilitate larger differences in evapotranspiration between land cover types, increasing the effect of forest cover on river flow. However, the effect of forest establishment on annual river flow is less sensitive to variable annual precipitation when the catchment is arid. The response of less arid catchments to changing precipitation is likely driven by larger changes in interception and subsequent evaporation. It is possible that variation in tree species ecophysiology and soil storage capacity have resulted in catchment aridity being a poor predictor of water limitation at the tree level. However, we are limited in our inferences by the small number of studies in catchments with a high aridity index. Despite the average effect of annual PET being not significantly different from zero, substantial variation in the effect of annual PET was present between catchments, with the magnitude of responses generally comparable with those to variable annual precipitation. This result corresponds with those of the single catchment analysis, where multiple catchments showed a significant positive or negative effect of annual PET on river flow response, possibly also due to the variation in forest responses to water stress, regulation of transpiration and soil storage capacity.

### 4.2 Trajectory of river flow with forest age

We find that, after accounting for confounding drivers of river flow recovery such as reported clearing and annual climatic variation, signals of river flow recovery driven by forests age are rare. This suggests that generally, for up to the five decades following establishment, no substantial river flow recovery can be expected to occur after the initial decline. Where patterns of river flow recovery did occur, the timing and rate of recovery varied substantially. Whilst one catchment demonstrates a strong signal of recovery, it is confounded by a substantial decline in precipitation over the same period (Flamisell). The authors of both catchment studies recovering to above control flows (Flamisel and Escalo) highlight the role of reduced precipitation during the time series in driving observed river flow responses (Buendia, Batalla, Sabater, Palau, & Marcé, 2016). Prior discussions of river flow recovery primarily suggest decreasing stand leaf area index and increasing infiltration rates are responsible for increasing in groundwater level (Asbjornsen et al., 2011; Ellison et al., 2017; Ilstedt et al., 2016; Vertessy et al., 2001). However, primary studies that report changes in forest water use with age often use timescales (via space for time substitutions) that dramatically exceed those in this paper and in other catchment studies that report river flow recovery (Delzon & Loustau, 2005; Farley et al., 2005; Ilstedt et al., 2016; Vertessy et al., 2001). Although forest establishment can increase rates of ground water recharge as a result of increased infiltration (Ilstedt et al., 2016; Zhou et al., 2010), if human land use and soil erosion have been intensive, soil moisture storage capacity may have been sufficiently reduced to inhibit meaningful recovery (Bruijnzeel, 2004). Where soil erosion rates can be reduced to enable net soil genesis (Verheijen, Jones, Rickson, & Smith, 2009), future recovery of ground water levels may be possible. It is advisable that further investigation be carried out when a greater number of long‐term time series are available. Of the 43 catchments that qualified for inclusion in this study, 19 of them do not reach the mean forest age at which the modelled minimum in river flow response was reported.

We find that increasing river flow following forest establishment is also rare. Negligible or positive changes in river flow response to forest establishment have been reported by Zhang et al. (2017) and Beck et al. (2013) and may result from the presence of extreme soil degradation prior to forestation and hydrological dynamics in tropical catchments not captured in this analysis. A history of recent deforestation may also offset initial river flow declines, although such studies were excluded from this review. Here, all cases of increasing river flow were small in magnitude and could be due to residual error in catchment calibrations. Questions remain about how forest ageing processes are affected by species richness and management practices, both of which have undergone a shift in recent years along the principal objectives behind contemporary forestation.

### 4.3 Wider implications

Widespread afforestation, reforestation and spontaneous forest regeneration remain important to current and future endeavours to counter biodiversity loss and anthropogenic climate change. However, our study reinforces the findings of previous research, showing that forestation is associated with significant decreases in river flow at annual timescales. In many places where river flow has value for both economic activity and welfare (Meyer, 1997), this would constitute a notable ecosystem disservice, particularly given predicted decreases in precipitation reliability for many parts of the world (IPCC, 2014). Conversely, many would consider a reduction in annual river flow part of an effective environmental restoration programme to achieve historic conditions (Hjältén, Nilsson, Jørgensen, & Bell, 2016; Sterba, Mekotova, Krskova, Samsonova, & Harper, 1997), though determining whether reduced river flows are reflective of historical conditions is beyond the scope of this review. Rather than assert whether changes in river flow resulting from forestation would constitute an ecosystem service or disservice overall, we emphasize the potential importance of accounting for these changes in future forestation programmes so that forests may be accurately valued, to the benefit of local communities (Egginton et al., 2014). Whilst we found limited evidence for partial river flow recovery with forest age, we found that forestation on agricultural land results in a smaller reduction in river flow than on idle land, likely as a result of increased infiltration rates. Although catchments with lower MAP will experience smaller absolute declines in river flow following forest establishment, substantial changes in river flow can be expected as forests age and spread. When annual precipitation decreases, the absolute effect of forestation on river flow also decreases, and our research suggests that this effect will be largest in wetter catchments. The effect of changing annual PET is highly variable, independent of catchment aridity metrics. Where forests are highly responsive to annual precipitation, reduced rates of evapotranspiration will partially offset reduced precipitation inputs, which will be of particular importance to ecosystems and communities that are at risk from water limitation. Further research is required to determine how forest seasonality and annual precipitation interact on a sub‐annual basis and how these trends might differ in tropical ecosystems, for which the lack of data is a notable, considering the numerous initiatives for tropical forest restoration and importance of these projects for biodiversity.

## CONFLICT OF INTEREST

The authors have no conflicts of interest to declare.

## Data Availability

The data that support the findings of this study are openly available in the Environmental Information Data Centre (EIDC, https://catalogue.ceh.ac.uk/eidc/documents) at https://doi.org/10.5285/5baa5d91-d552-4fc6-8a8c-29ae45192d77.
