# Supplementary material for: Partial river flow recovery with forest age is rare in the decades following establishment
Source: Glob Chang Biol. 2020 Jan 19;26(3):1458–73. doi: 10.1111/gcb.14954 (PMC7079061; doi:10.1111/gcb.14954)
Supplement: Supplementary file 1 [file GCB-26-1458-s001.pdf]

Partial river flow recovery with forest age is rare in the decades following establishment

Running Title: River flow responses to forest establishment

Supplementary Material

Laura Bentley, David Anthony Coomes *

Department of Plant Sciences, University of Cambridge, Downing Street, Cambridge, CB2 3EA

*Corresponding author: 01223 333911 dac18@cam.ac.uk

S1. Summary of database studies

Table S1 A summary of all catchments incorporated in our database, including a reference to the relevant primary source. NR is not reported. SD is standard deviation, MPET is mean annual potential evapotranspiration. All other terms are defined in the main text.

Catchment Name	MAP (mm)	P SD (mm)	MPET (mm)	PET SD (mm)	Area (km2)	P from CRUTS4	Q_{Ci} corrected	Start Year	Study Duration	Source
Mabegondo	1490	500.6	896	48.5	0.107	No	No	1999	10	Suarez, Perez, & Soto (2014)
Pomba	1400	152.4	1127	17.1	8600	Yes	No	2000	5	dos R. Pereira, Martinez, da Silva, & Pruski (2016)
Manuel Diaz	1400	110.1	1252	24.9	2097	No	Yes	1994	14	Silveira & Alonso (2009)
Don Thomas	1208	542.8	1292	54.2	2.12	No	Yes	2007	8	Silveira, Gamazo, Alonso, & MartÃ-nez (2016)
Glencar	2597	0	528	0	0.76	No	No	2008	2	Lewis, Albertson, Zi, Xu, & Kiely (2013)
Severn	2213	384.9	569	29.5	NR	No	Yes	1970	7	Calder & Newson (1979)
Coalburn	1400	143.3	463	27.9	1.5	No	Yes	1974	36	Birkinshaw, Bathurst, & Robinson (2014)
Cwm	2019	146.2	470	44.1	2.89	No	Yes	1985	13	Hudson, Crane, & Robinson (1997)
Yangjiagou	523	106.2	874	37.4	0.92	No	No	1956	54	Peng, Tague, & Jia (2016)
Zi-Fang-Gully	550	153.7	970	43.8	8.72	No	No	1973	30	Wang, Xin, Williams, & Xu (2006)
Luoyugou	1533	112.4	767	24.4	73	No	No	1996	13	Zhao & Yu (2013)
Crawford	762	88.7	1108	29.2	698	No	Yes	1998	9	H. Li et al. (2012)

Table S1 continued.

Catchment Name	MAP (mm)	P SD (mm)	MPET (mm)	PET SD (mm)	Area (km2)	P from CRUTS4	Q_{Ci} corrected	Start Year	Study Duration	Source
Ganaraska	859	97.3	729	39.0	267	No	Yes	1951	57	Buttle (2011)
Canalda	620	182.5	925	46.1	65	No	No	1986	24	C. Buendia et al. (2016)
Goc Mountain MIII	793	106.2	858	43.5	0.0843	Yes	No	1980	16	Ristic & Macan (1997)
Goc Mountain MI	793	106.2	858	43.5	0.0786	Yes	No	1980	16	Ristic & Macan (1997)
Escalo	1123	191.2	905	48.8	438	No	No	1988	22	Cristina Buendia et al. (2016)
Flamisell	1050	158.8	947.8	47.1	345	No	No	1988	22	Cristina Buendia et al. (2016)
Bartin	989	155.1	903	28.8	287	No	No	2006	20	Öztürk, Copty, & Saysel (2013)
Zhangjiashan	517	102.5	870	35.6	41800	No	No	1971	39	Chang et al. (2016)
Zhuangtou	544	100.6	950	43.0	25645	No	No	1971	39	Chang et al. (2016)
Shimen	1542	219.0	870	13.8	15307	No	Yes	2007	3	Zheng, Sun, & Yan (2014)
Lianshui	1600	229.5	963.3	24.9	579	No	Yes	1984	26	S. Li, Xu, & Sun (2014)
Moutere C4	1051	44.7	763	16.6	0.0271	Yes	No	1983	5	(Smith & Scott, 1992)

Table S1 continued.

Catchment Name	MAP (mm)	P SD (mm)	MPET (mm)	PET SD (mm)	Area (km2)	P from CRUTS4	Q_{Ci} corrected	Start Year	Study Duration	Source
Glenmorgan	1535	294.9	1385	23.9	0.3189	No	No	1972	10	Samraj, Sharda, Chinnamani, Lakshmanan, & Haldorai (1988)
Tarawera	1475	179.9	771	11.8	906	No	Yes	1964	18	Dons (1986)
Batalling Creek	610	22.7	1530	31.9	16.6	Yes	No	1997	3	Hickel & Zhang (2007)
Lambrechtsbos B	1145	218.3	1405	36.9	0.656	No	No	1965	32	Scott et al. (2000)
Bosboukloof	1127	197.5	1405	36.2	2.009	No	No	1946	33	Scott et al. (2000)
Biesievlei	1298	247.9	1405	35.6	0.272	No	No	1948	36	Scott et al. (2000)
Tierkloof	1319	217.6	1410	31.4	1.572	No	No	1956	41	Scott et al. (2000)
Lambrechtsbos A	1127	197.8	1415.8	35.0	0.312	No	No	1972	19	Scott et al. (2000)
Glendhu GH2	980	157.4	649.8	17.9	3.1	No	No	1987	27	Fahey & Payne (2017)
Westfalia D	1253	390.7	1310	41.7	0.396	No	No	1983	12	Scott et al. (2000)
Cathedral Peak III	1446	26.3	116.5	20.7	1.42	No	No	1959	4	Scott et al. (2000)
Mokobulaan A	1166	182.1	1125.5	35.2	0.262	No	No	1969	16	Scott et al. (2000)

Table S1 continued.

Catchment Name	MAP (mm)	P SD (mm)	MPET (mm)	PET SD (mm)	Area (km2)	P from CRUTS4	Q_{Ci} corrected	Start Year	Study Duration	Source
Mokobulaan B	1180	222.9	1131	33.9	0.346	No	No	1971	21	Scott et al. (2000)
Cathedral peak II	1399	274.1	1144	26.8	1.9	No	No	1951	30	Scott et al. (2000)
Tinana	1038	312.2	1547	44.1	1174	No	Yes	1971	28	H. Li, Zhang, Vaze, & Wang, (2012)
Gatum FC	618	167.3	1279	47.1	3.4	No	No	2010	3	Adelana et al. (2015)
Darlot	700	94.1	1180	48.9	760	No	Yes	1997	9	H. Li et al. (2012)
Pine Creek	775	157.7	1236	27.8	3.2	No	No	1998	6	Hickel & Zhang (2007)
Traralgon Creek	966	159.5	1069	31.3	87	Yes	No	1993	4	Hickel & Zhang, (2007)

S2. Data Extraction Table

Table S2 Catchment meta-data data extracted from each primary data source, along with relevant factor levels.

Name	Variable Description	Notes
**Source_ID**	Unique id for data source	
**Catchment ID**	Unique id for catchment	
**Catchment Name**	Name of forested catchment	
**Area.km2**	Area of forested catchment (km^2)	
**Lat**	Latitude of forested catchment (decimal degrees to given accuracy)	
**Lon**	Longitude of forested catchment (decimal degrees to given accuracy)	
**MAP**	Mean Annual precipitation (mm)	Where annual rainfall in mm is given for a parent catchment rather than the relevant sub catchment only, this value will be accepted, as long as it is standardised for area.
**Prior_LC**	Land cover of forested catchment before treatment: Grassland, Shrubland, Other	Agricultural areas assumed grassland unless specified otherwise
**Prior_LU**	Agriculture, Idle, Other	
**Establishment**	Passive, Active (using natural/planted definition from FAO http://www.fao.org/forestry/plantedforests/67504/en/), or mixed	
**Forest_Type_BN**	Forest Type: Broadleaf, Conifer, Other	Other category is a combination of mixed and unknown.
**Forest_Type_ED**	Forest Type: Evergreen Broadleaf, Evergreen Conifer, Deciduous or Mixed, Unknown	
**Hydro_Regime**	Hydrological Regime: Rain or Snow dominated or mix	If no mention is made of snow in the study paper, it is assumed that precipitation was rain dominated
**Tree_Age**	Multiple discrete planting, Continuous establishment	
**Experiment_Type**	Single or paired catchment experiment	
**Graphically_Extracted**	Y – River flow, forest cover or precipitation data (if required) was extracted from a figure.<br>N – Data was extracted from text	
**Modified_Data**	Y - Data reported in the paper has been modified. This is to control for a difference in rainfall between control and forested catchment.<br>N – No modification	Does not refer to unit conversion.

**Data_Set_Duration**	Number of years covered by river flow data set, including and baseline and calibration data	Exclude data gaps
**T_Duration**	Number of years of river flow data in forested catchment	
**C_Duration**	Number of years of river flow data in control catchment	
**Historically_forested**	Yes – Historic forest cover is reported, or forest establishment is described as reforestation, No – Catchment is reported to have not been forested historically, Not reported – no information about forest history is provided.	
**QuasiPaired**	Yes – A calibration had not been applied to control data prior to extraction, No – A calibration had been applied to control data prior to extraction	Refers to the form of the data available for extraction and may not appropriately describe the full methodology of the relevant study

S3. Explanatory Factor Associations

Table S3 Spatial variables which are confounded with one another, tested with Fisher's exact test where both variables are discrete, ANOVA where one variable was discrete and the other continuous.

Variable 1	Variable 2	p value	Holm corrected p value	Confounded
Prior_LU	Prior_LC	< 0.001	< 0.001	Y
Prior_LU	Forest_Type_BN	0.048	0.385	N
Prior_LU	Historically_forested	0.035	0.318	N
Prior_LU	Establishment	0.624	1.000	N
Prior_LU	QuasiPaired	0.009	0.093	N
Prior_LC	Forest_Type_BN	0.115	0.664	N
Prior_LC	Historically_forested	0.054	0.385	N
Prior_LC	Establishment	0.111	0.664	N
Prior_LC	QuasiPaired	0.001	0.014	Y
Forest_Type_BN	Historically_forested	0.193	0.771	N
Forest_Type_BN	Establishment	0.000	0.006	Y
Forest_Type_BN	QuasiPaired	0.002	0.027	Y
Historically_forested	Establishment	0.842	1.000	N
Historically_forested	QuasiPaired	< 0.001	0.005	Y
Establishment	QuasiPaired	0.675	1.000	N
Prior_LU	Prior_LC	< 0.001	< 0.001	Y
MAP	Prior_LU	0.684	1	N
MAP	Prior_LC	0.822	1	N
MAP	Forest_Type_BN	0.082	1	N
MAP	Historically_forested	0.471	1	N
MAP	Establishment	0.304	1	N
MAP	QuasiPaired	0.057	1	N
FC_PD_Between	Prior_LU	0.150	1	N
FC_PD_Between	Prior_LC	0.161	1	N
FC_PD_Between	Forest_Type_BN	< 0.001	<0.001	Y
FC_PD_Between	Historically_forested	0.110	1	N
FC_PD_Between	Establishment	0.039	0.786	N
FC_PD_Between	QuasiPaired	0.005	0.105	N
CRUTS4_AI_Between	Prior_LU	0.688	1	N
CRUTS4_AI_Between	Prior_LC	0.357	1	N
CRUTS4_AI_Between	Forest_Type_BN	0.142	1	N
CRUTS4_AI_Between	Historically_forested	0.057	1	N
CRUTS4_AI_Between	Establishment	0.631	1	N
CRUTS4_AI_Between	QuasiPaired	0.105	1	N
CRUTS4_PET_Between	Prior_LU	0.593	1	N
CRUTS4_PET_Between	Prior_LC	0.018	0.377	N
CRUTS4_PET_Between	Forest_Type_BN	0.098	1	N
CRUTS4_PET_Between	Historically_forested	< 0.001	0.006	Y
CRUTS4_PET_Between	Establishment	0.670	1	N
CRUTS4_PET_Between	QuasiPaired	0.054	1	N

Table S4 Results of Tukey HSD test for the association between FC_B and FT.

Comparison	difference	2.5 percent CI	97.5 percent CI	p value
Conifer-Broadleaf	18.657	-4.905	42.218	0.144
Other-Broadleaf	-39.510	-65.715	-13.304	0.002
Other-Conifer	-58.166	-82.304	-34.029	<0.001

Table S5 Pearson's correlation tests for continuous spatial explanatory variables.

Variable 1	Variable 2	Pearson's r	P value	Correlated
MAP	FC_PD_Between	0.251	0.1051	N
MAP	CRUTS4_AI_Between	0.636	< 0.0001	Y
MAP	CRUTS4_PET_Between	-0.311	0.0425	Y
FC_PD_Between	CRUTS4_AI_Between	0.117	0.4545	N
FC_PD_Between	CRUTS4_PET_Between	-0.012	0.9397	N
CRUTS4_AI_Between	CRUTS4_PET_Between	-0.679	< 0.0001	Y

Table S6 Pearson's correlation tests for associations between climatic variability and mean climate.

Variable 1	Variable 2	Pearson's r	P value
MAP	P range	0.174	0.262
MAP	P SD	0.240	0.121
Mean PET	PET range	0.153	0.328
Mean PET	PET SD	0.244	0.114

S4. NLME Catchment level coefficients

Table S7 Catchment level estimates for coefficient values of a, b, c and d in equation 10.

Name	a	b	c	d
Westfalia D	-31.734	0.612	-0.198	0.438
Cathedral Peak III	-13.801	0.090	-0.001	-0.216
Lambrechtsbos B	-8.670	0.061	0.033	-0.215
Mokobulaan A	-21.005	0.353	-0.059	1.397
Mokobulaan B	-11.202	0.149	-0.017	0.201
Cathedral peak II	-26.380	0.486	-0.178	-0.141
Bosboukloof	-4.854	0.024	0.034	-0.575
Biesievlei	-22.813	0.358	-0.108	-0.269
Tierkloof	-3.770	-0.003	0.039	-0.718
Lambrechtsbos A	-18.797	0.311	-0.066	0.198
Severn	-20.819	0.155	-0.037	0.464
Tarawera	-3.180	-0.008	-0.061	-0.189
Glenmorgan	-14.440	0.198	-0.041	0.289
Gatum FC (Gatum PC)	-4.492	0.201	-0.025	0.035
Lianshui	-8.988	0.175	-0.079	0.412
Mabegondo	-21.615	0.350	-0.167	-0.161
Coalburn	1.491	-0.194	-0.012	-1.341
Shimen	-7.982	0.175	-0.084	-0.142

Glencar	-31.881	0.001	-0.139	-0.281
Glendhu GH2 (Glendhu GH1)	-18.050	0.411	-0.146	0.843
Bartin	-7.109	0.209	-0.066	-0.068
Luoyugou	-11.168	0.261	-0.067	-0.003
Crawford	-5.779	0.185	-0.033	-0.062
Darlot	-5.939	0.189	-0.028	-0.012
Tinana	-6.397	0.223	-0.072	-0.379
Ganaraska (Duffins Creek)	0.474	0.029	-0.013	-0.400
Manuel Diaz	-13.980	0.357	-0.117	0.378
Batalling Creek	-2.800	0.126	0.007	-0.142
Pine Creek	-2.514	0.129	-0.029	-0.138
Traralgon Creek	0.328	0.004	0.030	-0.513
Zi-Fang-Gully	-3.874	0.196	-0.025	-0.030
Goc Mountain MIII (Goc Mountain MII)	-4.265	0.067	0.001	-0.085
Goc Mountain MI (Goc Mountain MII)	-6.262	0.128	-0.032	-0.198
Cwm (Delyn)	-26.547	0.297	-0.332	-0.175
Don Thomas (La Cantera)	-19.388	0.434	-0.145	0.557
Moutere C4 (Moutere C2)	-4.256	0.120	-0.104	-0.132
Zhangjiashan	-3.359	0.150	-0.013	-0.099
Zhuangtou	-2.563	0.126	0.001	-0.087
Yangjiagou (Dongzhuanggou)	-0.734	0.028	0.050	0.109
Escalo	-0.987	0.009	-0.011	-0.814
Flamisell	-5.377	0.071	0.011	0.150
Pomba	-4.253	0.139	-0.055	-0.123
Canalda	-6.355	0.208	-0.057	0.004

S5. Single Catchment Models

Table S8 Catchment level estimates for coefficient values of a, b, c, d, e, f, h and k where applicable in equations 2 or 3 respectively. Coefficient g *was dropped from all models.*

Catchment	a	b	h	k	c	d	e	f
Tinana	-19.3	1	-	-	-	-	-	-
Gatum FC	-20.1	2.33	-	-	-	-	-	-
Darlot	-	-	-37.9	0.745	-0.0593	0.0012	-0.0449	-
Traralgon Creek	33.9	-1.39	-	-	-	-	-	-
Pine Creek	-2.16	-	-	-	0.152	-0.000217	-	-
Crawford	-	-	-50.9	0.655	-0.0577	0.00167	-0.22	0.00353
Batalling Creek	1.88	-0.114	-	-	-	-	-	-
Pomba	0.852	-0.608	-	-	-	-	-	-
Ganaraska	3.56	-0.0336	-	-	-	-	-	-
Lianshui	-31.2	1.9	-	-	-	-	0.842	0.0747
Shimen	-3.81	-	-	-	-0.283	-	-	-
Yangjiagou	-0.191	0.00203	-	-	-0.0401	-0.000128	-	-
Zi-Fang-Gully	-	-	-10.7	-0.474	-0.022	-	-0.0295	0.000509
Luoyugou	-	-	-	-	0.209	0.000193	-	-
Zhangjiashan	-	-	-16.2	-1.91	-0.0289	0.000158	-0.123	-
Zhuangtou	-0.03	-0.0217	-	-	0.0245	-0.0000551	-0.0669	0.000656
Mabegondo	-	-	-302	-2.03	-0.174	-	-	-
Escalo	-13.7	2.11	-	-	-	-	-	-
Flamisell	-19.9	2.6	-	-	-	-	0.211	-0.0107
Canalda	-	-	-30.6	-1.86	-0.0996	-	-	-
Severn	-	-	-607	-2.98	0.143	-0.000596	1.11	0.0691
Coalburn	9.57	-0.43	-	-	-	-	-3.04	0.0329
Cwm	-	-	-272	-0.818	-1.23	0.00237	-1.55	-0.0826
Glenmorgan	-	-	-192	-2.09	-0.034	-	-	-
Glencar	-29.7	-	-	-	-	-	-	-
Tarawera	-	-	-4.35	-0.815	0.00791	-0.000025	-	-
Moutere C4	-4.43	-0.497	-	-	-	-	-	-
Glendhu GH2	-	-	-304	-2.15	-	-	2.71	0.0571
Goc Mountain MIII	-	-	-1.16	-2.21	-0.00539	0.0000437	-0.0116	-0.0001
Goc Mountain MI	-0.0731	0.00113	-	-	-	-	-	-
Bartin	-	-	-171	-3.25	-0.0622	-	-	-
Manuel Diaz	-	-	-	-	5.06	0.02	-	-
Don Thomas	-36.3	1.77	-	-	-	-	2.56	-0.0331
Westfalia D	-	-	-570	-1.81	-0.2	-	-	-
Cathedral Peak III	84.7	-19.5	-	-	-	-	-	-
Mokobulaan A	-	-	-391	-1.84	-	-	2.89	-
Mokobulaan B	-	-	-243	-2.37	-	-	0.838	0.028
Cathedral peak II	-	-	-1090	-3.75	-0.118	-	-1.24	-
Lambrechtsbos B	-2.21	-0.0882	-	-	-	-	-0.172	0.000242
Bosboukloof	-16.2	0.326	-	-	0.137	0.000244	-0.675	0.0109
Biesievlei	-	-	-368	-2.58	-0.124	-	-0.163	0.0123
Tierkloof	-	-	-126	-1.94	0.142	-	-	-
Lambrechtsbos A	-	-	-271	-1.55	-	-	-	-

Table S9 Single catchment alternate model AICs

Catchment	Linear Model selection AIC	Non-linear model selection AIC	AIC Difference	Preferred Structure
Tinana	320.65	-	-	Polynomial
Gatum FC (Gatum PC)	20.28	-	-	Polynomial
Darlot	57.44	59.32	-1.89	Asymptotic
Traralgon Creek	31.50	-	-	Polynomial
Pine Creek	18.25	-	-	Linear
Crawford	63.53	58.11	-5.42	Asymptotic
Batalling Creek	10.64	-	-	Polynomial
Pomba	28.18	-	-	Polynomial
Ganaraska (Duffins Creek)	532.94	-	-	Polynomial
Lianshui	171.64	-	-	Polynomial
Shimen	27.94	-	-	Linear
Yangjiagou (Dongzhuanggou)	297.77	-	-	Polynomial
Zi-Fang-Gully	176.68	165.94	-10.73	Asymptotic
Luoyugou	107.11	-	-	Linear
Zhangjiashan	275.91	275.83	-0.08	Asymptotic
Zhuangtou	240.10	-	-	Polynomial
Mabegondo	120.76	119.30	-1.46	Asymptotic
Escalo	272.14	-	-	Polynomial
Flamisell	234.26	-	-	Polynomial
Canalda	202.87	201.12	-1.74	Asymptotic
Severn	87.87	87.59	-0.28	Asymptotic
Coalburn	307.50	-	-	Polynomial
Cwm (Delyn)	90.21	89.79	-0.42	Asymptotic
Glenmorgan	113.54	115.39	-1.85	Asymptotic
Glencar	29.26	-	-	Linear
Tarawera	64.66	66.83	-2.17	Asymptotic
Moutere C4 (Moutere C2)	51.34	-	-	Polynomial
Glendhu GH2 (Glendhu GH1)	296.03	299.58	-3.55	Asymptotic
Goc Mountain MIII (Goc Mountain MII)	43.29	47.51	-4.22	Asymptotic
Goc Mountain MI (Goc Mountain MII)	39.59	-	-	Polynomial
Bartin	136.77	138.10	-1.33	Asymptotic
Manuel Diaz	39.13	-	-	Linear
Don Thomas (La Cantera)	100.94	-	-	Polynomial
Westfalia D	95.86	95.30	-0.56	Asymptotic
Cathedral Peak III	26.31	-	-	Polynomial
Mokobulaan A	136.97	136.78	-0.20	Asymptotic

Mokobulaan B	119.41	119.93	-0.52	Asymptotic
Cathedral peak II	360.60	359.45	-1.15	Asymptotic
Lambrechtsbos B	127.13	-	-	Polynomial
Bosboukloof	372.19	-	-	Polynomial
Biesievlei	400.83	404.69	-3.85	Asymptotic
Tierkloof	497.13	497.39	-0.26	Asymptotic
Lambrechtsbos A	202.25	211.15	-8.90	Asymptotic

S6. Impact of including data where $Q_{Ai} = 0$ on recovery analysis

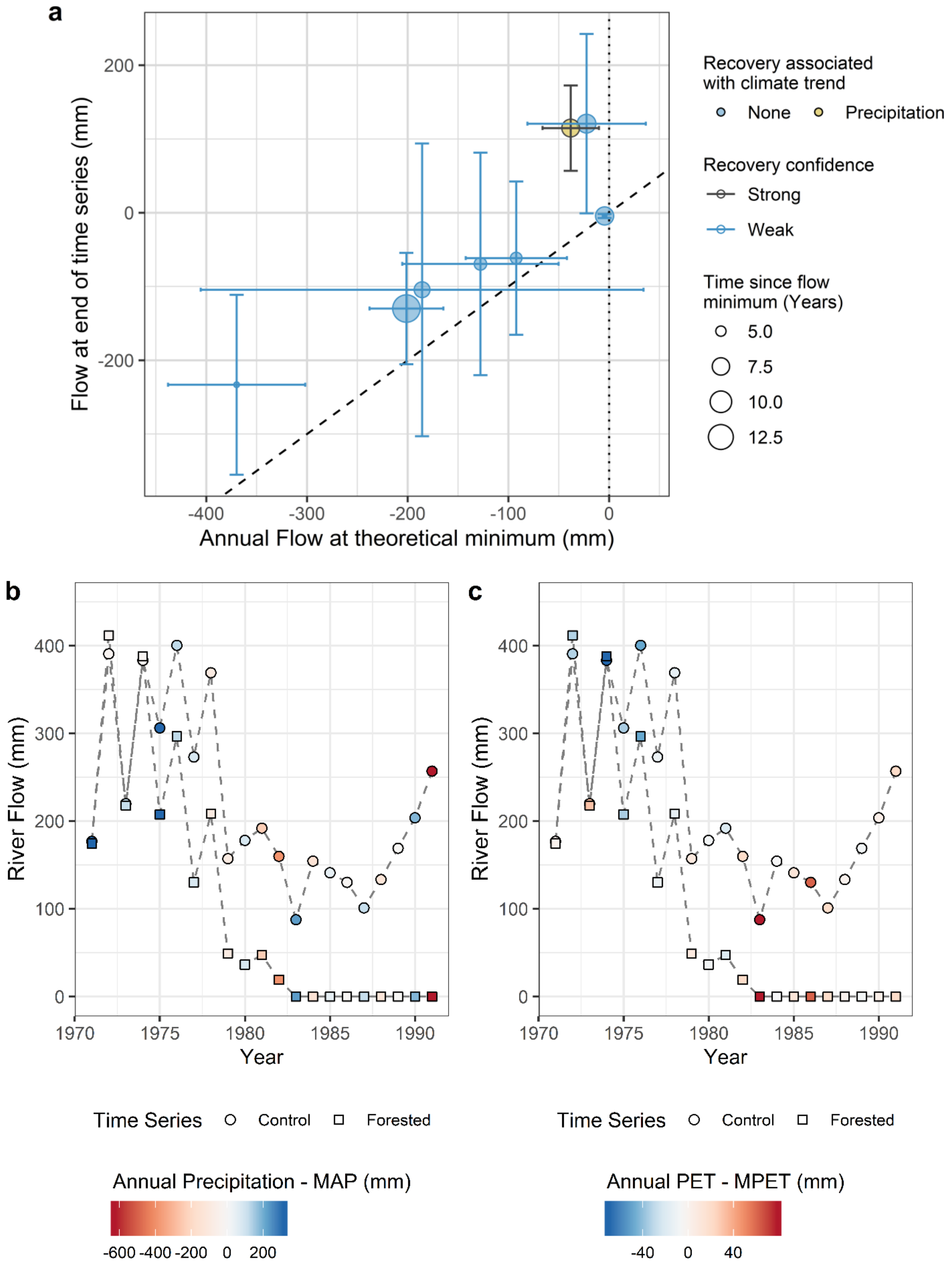

Figure S1 ***(a)*** *Magnitude of river flow recovery observed between minimum flow and final data point, with data points where $Q_{Ai} = 0$ are included. A time series of absolute river flow in both control and afforestation datasets for catchment Mokobulaan B, illustrating the change in control flow that is not associated with a change in annual precipitation* ***(b)*** *or annual PET* ***(c)*** *within this catchment.*

S7. Annual Precipitation Agreement

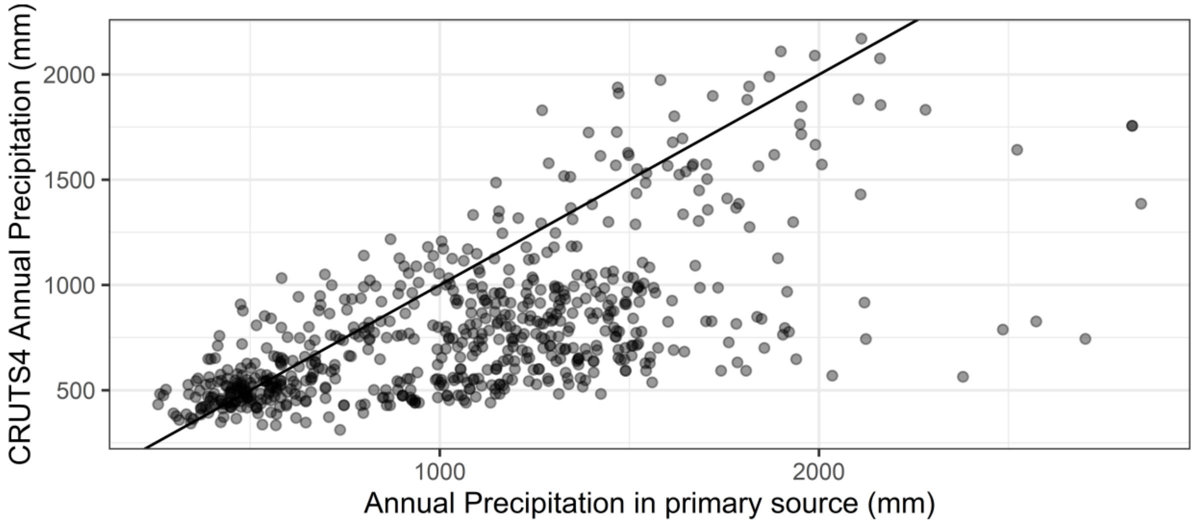

Figure S2 Correlation between annual precipitation reported in primary literature and extracted from CRUTS4.

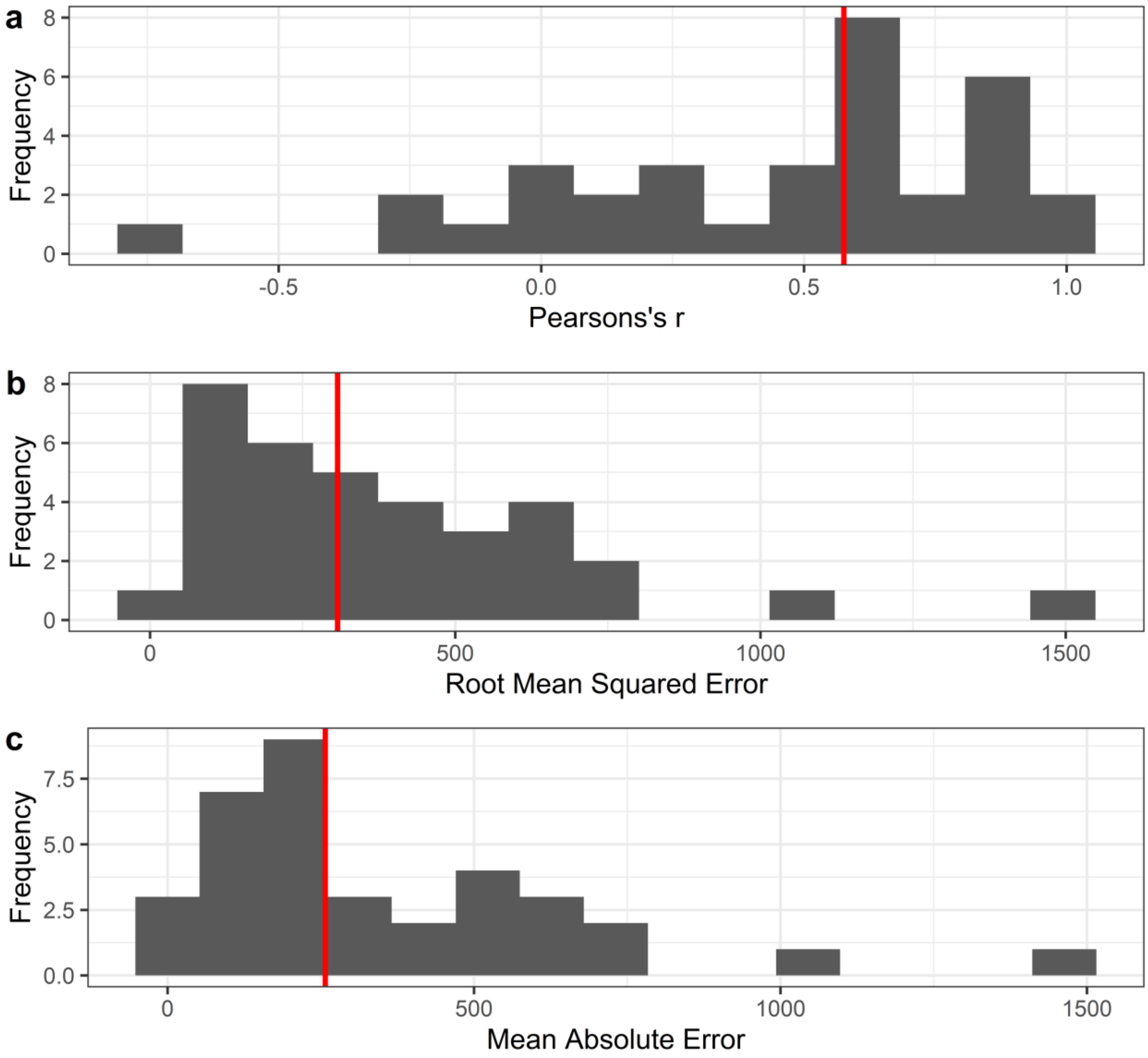

Figure S3 Distribution of agreement between reported annual precipitation and CRUTS4 annual precipitation at the catchment scale, measured by ***(a)*** *Pearson's r,* ***(b)*** *Root mean squared error and* ***(c)*** *Mean Absolute Error. Median values a shown by vertical line.*

S8. Results of repeat analyses

8.1 – Incorporating *PLC* in equation of *a*

The final model is shown in equations 16-20. A summary of model coefficients is given in Table S10 and S11. All coefficients are defined in the main text. Coefficients h, m and o are significant at the $p < 0.05$ level following and ANOVA F test using marginal sum of squares. All other coefficients reported $p > 0.05$. AIC of the final model was 8761.8

$$Q_i = a\, Age_i + b\, Age_i^{\,2} + c\, P_{Ti} + d\, PET_{Ti} + \varepsilon \tag{16}$$

Where

$$a \sim N(e + f\, MAP + g\, FC_S + h\,(MAP * FC),\ \sigma_1^2 = 64.5) \tag{17}$$

$$b \sim N(m, \sigma_2^2 = 0.0376) \tag{18}$$

$$c \sim N(n + o\, Aridity, \sigma_3^2 = 0.00602) \tag{19}$$

$$d \sim N(r, \sigma_3^2 = 0.327) \tag{20}$$

Table S10 Correlation matrix between random effects

	σ_2^2	σ_3^2	σ_4^2
σ_1^2	-0.998	0.729	-0.510
σ_2^2		-0.722	0.442
σ_3^2			-0.187

Table S11 Mixed effect model fixed effect coefficient values.

**Response variable**	**Coefficient**	**Value**	**Standard Error**
a	*e*	-3.33	3.6
a	*f*	-0.00202	0.0033
a	*g*	0.0828	0.053
a	*h*	-0.000130	0.000052
b	*m*	0.143	0.047
c	*n*	0.0155	0.33
c	*o*	-0.071	0.29
d	*r*	-0.058	0.12

8.2 – Incorporating *FT* in equation of a

The final model is shown in equations 21-25. A summary of model coefficients is given in Table S12 and S13. Coefficient *e* is the intercept where MAP = 0 and *FT* = broadleaf. All other coefficients are defined in the main text. Coefficients f, g, m and o are significant at the $p < 0.05$ level following and ANOVA F test using marginal sum of squares. All other coefficients reported $p > 0.05$. AIC of the final model was 8761.0.

$$Q_i = a\, Age_i + b\, Age_i^{\,2} + c\, P_{Ti} + d\, PET_{Ti} + \varepsilon \tag{21}$$

Where $a \sim N(e + f\ MAP + gFT,\ \sigma_1^2 = 75.7)$ (22)

$b \sim N(m, \sigma_2^2 = 0.0751)$ (23)

$c \sim N(n + o\ Aridity, \sigma_3^2 = 0.00529)$ (24)

$d \sim N(r, \sigma_3^2 = 0.297)$ (25)

Table S12 Correlation matrix between random effects

	σ_2^2	σ_3^2	σ_4^2
σ_1^2	-0.993	0.757	-0.538
σ_2^2		-0.708	0.482
σ_3^2			-0.202

Table S13 Mixed effect model fixed effect coefficient values.

Response variable	Coefficient	Value	Standard Error
a	*e -broadleaf*	-0.484	2.4
a	*f*	-0.00926	0.0016
a	*g -conifer*	-0.349	1.4
a	*g - other*	4.22	1.4
b	*m*	0.165	0.049
c	*n*	0.0109	0.32
c	*o*	-0.0679	0.28
d	*r*	-0.0746	0.12

8.3 – Incorporating aridity in equation of a

The final model is shown in equations 26-30. A summary of model coefficients is given in Table S14 and S15. Coefficient e is the intercept of a where MAP and FC are 0 and HF (historically forested) is No. All other coefficients are defined in the main text. Coefficients e, h, k, m, and o are significant at the $p < 0.05$ level following and ANOVA F test using marginal sum of squares. All other coefficients reported $p > 0.05$. AIC of the final model was 8775.6

$$Q_i = a\ Age_i + b\ Age_i^{\ 2} + c\ P_{Ti} + d\ PET_{Ti} + \varepsilon \quad (26)$$

Where $a \sim N(e + f\ Aridity + g\ FC_S + h\ (\text{Aridity} * FC) + kHF,\ \sigma_1^2 = 90.1)$ (27)

$b \sim N(m, \sigma_2^2 = 0.0412)$ (28)

$c \sim N(n + o\ Aridity, \sigma_3^2 = 0.00579)$ (29)

$d \sim N(r, \sigma_3^2 = 0.366)$ (30)

Table S14 Correlation matrix between random effects

	σ_2^2	σ_3^2	σ_4^2
σ_1^2	-0.998	0.577	-0.542
σ_2^2		-0.547	0.425
σ_3^2			-0.069

Table S15 Mixed effect model fixed effect coefficient values.

Response variable	Coefficient	Value	Standard Error
a	*e - No*	-14.2	3.3
a	*f*	2.37	2.8
a	*g*	0.0645	0.040
a	*h*	-0.0977	0.037
a	*k - Yes*	4.61	2.3
a	*k -Unreported*	7.10	1.2
b	*m*	0.145	0.046
c	*n*	0.0199	0.034
c	*o*	-0.0728	0.030
d	*r*	-0.0254	0.12

8.4 – Incorporating mean PET (MPET) in equation of a

The final model is shown in equations 31-35. A summary of model coefficients is given in Table S16 and S17. Coefficient e is the intercept of a where MPET = 0 and HF (historically forested) = No. All other coefficients are defined in the main text. Coefficients e, f, g, k, m, and o are significant at the $p < 0.05$ level following and ANOVA F test using marginal sum of squares. All other coefficients reported $p > 0.05$. AIC of the final model was 8777.9.

$$Q_i = a\,Age_i + b\,{Age_i}^2 + c\,P_{Ti} + d\,PET_{Ti} + \varepsilon \tag{31}$$

Where
$$a \sim N(e + f\,MPET + g\,FC_S + k\,HF,\ \sigma_1^2 = 108) \tag{32}$$

$$b \sim N(m, \sigma_2^2 = 0.0582) \tag{33}$$

$$c \sim N(n + o\,Aridity, \sigma_3^2 = 0.00602) \tag{34}$$

$$d \sim N(r, \sigma_3^2 = 0.339) \tag{35}$$

Table S16 Correlation matrix between random effects

	σ_2^2	σ_3^2	σ_4^2
σ_1^2	-0.997	0.732	-0.388
σ_2^2		-0.709	0.330
σ_3^2			-0.175

Table S17 Mixed effect model fixed effect coefficient values.

Response variable	Coefficient	Value	Standard Error
a	*e - No*	-25.3	5.3
a	*f*	0.0104	0.0033
a	*g*	-0.0383	0.014
a	*k - Yes*	6.26	2.8
a	*k -Unreported*	10.7	2.0
b	*m*	0.201	0.051
c	*n*	0.0110	0.033
c	*o*	-0.0668	0.030
d	*r*	-0.0332	0.13

S9. Sensitivity analyses

9.1 – Excluding catchments with corrected Q_{Ci}

The final model is shown in equations 36-40. A summary of model coefficients is given in Table S18 and S19. All coefficients are defined in the main text. Coefficients h, l, m and n are significant at the $p < 0.05$ level following and ANOVA F test using marginal sum of squares. All other coefficients reported $p > 0.05$. AIC of the final model was 7532.7. The data set contained 663 points over 36 catchments.

$$Q_i = a\,Age_i + b\,Age_i^{\,2} + c\,P_{Ti} + d\,PET_{Ti} + \varepsilon \quad (36)$$

Where

$$a \sim N(e + f\,MAP + g\,FC_S + h\,(MAP * FC) + lPLU,\ \sigma_1^2 = 8.86) \quad (37)$$

$$b \sim N(m, \sigma_2^2 = 0.227) \quad (38)$$

$$c \sim N(n, \sigma_3^2 = 0.0756) \quad (39)$$

$$d \sim N(r, \sigma_3^2 = 0.627) \quad (40)$$

Table S18 Correlation matrix between random effects

	σ_2^2	σ_3^2	σ_4^2
σ_1^2	-0.997	0.584	-0.552
σ_2^2		-0.561	0.499
σ_3^2			-0.232

Table S19 Mixed effect model fixed effect coefficient values.

Response variable	Coefficient	Value	Standard Error
a	*e - Agriculture*	-7.04	4.7
a	*f*	0.000376	0.0037
a	*g*	0.110	0.77
a	*h*	-0.000142	0.000062
a	*l - Idle*	-2.59	1.2
a	*l - Other*	1.71	2.3
b	*m*	0.194	0.061
c	*n*	-0.0524	0.019
d	*r*	-0.0723	0.14

9.2 – Only catchments with corrected Q_{Ci}

The final model is shown in equations 41-45. A summary of model coefficients is given in Table S20 and S21. All coefficients are defined in the main text. Coefficients g, h, and o are significant at the p < 0.05 level following and ANOVA F test using marginal sum of squares. All other coefficients reported p > 0.05. AIC of the final model was 1235.2. The dataset contained 107 points over 7 catchments.

$$Q_i = a\,Age_i + b\,Age_i^2 + c\,P_{Ti} + d\,PET_{Ti} + \varepsilon \quad (41)$$

Where
$$a \sim N(e + f\,MAP + g\,FC_S + h\,(MAP * FC),\ \sigma_1^2 = 5.03) \quad (42)$$

$$b \sim N(m, \sigma_2^2 = 0.119) \quad (43)$$

$$c \sim N(n + o\,Aridity, \sigma_3^2 = 0.0760) \quad (44)$$

$$d \sim N(r, \sigma_3^2 = 0.273) \quad (45)$$

Table S20 Correlation matrix between random effects

	σ_2^2	σ_3^2	σ_4^2
σ_1^2	-0.999	0.992	-0.992
σ_2^2		-0.993	0.993
σ_3^2			-0.993

Table S21 Mixed effect model fixed effect coefficient values.

Response variable	Coefficient	Value	Standard Error
a	*e*	-24.0	13
a	*f*	0.0258	0.015
a	*g*	0.458	0.20
a	*h*	-0.000613	0.00024
b	*m*	0.101	0.072
c	*n*	0.119	0.10
c	*o*	-0.142	0.06
d	*r*	0.0364	0.24

References

Adelana, S. M., Dresel, P. E., Hekmeijer, P., Zydor, H., Webb, J. A., Reynolds, M., & Ryan, M. (2015). A comparison of streamflow, salt and water balances in adjacent farmland and forest catchments in south-western Victoria, Australia. *Hydrological Processes*, *29*(6), 1630–1643. https://doi.org/10.1002/hyp.10281

Birkinshaw, S. J., Bathurst, J. C., & Robinson, M. (2014). 45 years of non-stationary hydrology over a forest plantation growth cycle, Coalburn catchment, Northern England. *Journal of Hydrology*, *519*(Part A), 559–573. https://doi.org/10.1016/j.jhydrol.2014.07.050

Buendia, C.., Bussi, G., Tuset, J., Vericat, D., Sabater, S., Palau, A., & Batalla, R. J. (2016). Effects of afforestation on runoff and sediment load in an upland Mediterranean catchment. *Science of the Total Environment*, *540*, 144–157. https://doi.org/10.1016/j.scitotenv.2015.07.005

Buendia, Cristina, Batalla, R. J., Sabater, S., Palau, A., & Marcé, R. (2016). Runoff Trends Driven by Climate and Afforestation in a Pyrenean Basin. *Land Degradation and Development*, *27*(3), 823–838. https://doi.org/10.1002/ldr.2384

Buttle, J. M. (2011). Streamflow response to headwater reforestation in the Ganaraska River basin, southern Ontario, Canada. *Hydrological Processes*, *25*(19), 3030–3041. https://doi.org/10.1002/hyp.8061

Calder, I. R., & Newson, M. D. (1979). Land-Use and Upland Water Resources in Britain-a Strategic Look. *Journal of the American Water Resouces Association*, *15*(6), 1628–1639.

Chang, J., Li, Y., Wei, J., Wang, Y., & Guo, A. (2016). Dynamic changes of sediment load and water discharge in the Weihe River, China. *Environmental Earth Sciences*, *75*(12), 1042. https://doi.org/10.1007/s12665-016-5841-9

Dons, A. (1986). The effect of large-scale afforestation on Tarawera river flows. *Journal of Hydrology (New Zealand)*, *25*(2), 61–73.

dos R. Pereira, D., Martinez, M. A., da Silva, D. D., & Pruski, F. F. (2016). Hydrological simulation in a basin of typical tropical climate and soil using the SWAT Model Part II: Simulation of hydrological variables and soil use scenarios. *Journal of Hydrology: Regional Studies*, *5*, 149–163. https://doi.org/10.1016/j.ejrh.2015.11.008

Fahey, B., & Payne, J. (2017). The Glendhu experimental catchment study, upland east Otago, New Zealand: 34 years of hydrological observations on the afforestation of tussock grasslands. *Hydrological Processes*, *31*(16), 2921–2934. https://doi.org/10.1002/hyp.11234

Hickel, K., & Zhang, L. (2007). Predicting Afforestation Impacts on Monthly Streamflow. *MODSIM 2007 International Congress on Modelling and Simulation*, 2527–2533.

Hudson, J. A., Crane, S. B., & Robinson, M. (1997). The impact of the growth of new plantation forestry on evaporation and streamflow in the Llanbrynmair catchments. *Hydrology and Earth System Sciences*, *1*(3), 463–475.

Lewis, C., Albertson, J., Zi, T., Xu, X., & Kiely, G. (2013). How does afforestation affect the hydrology of a blanket peatland? A modelling study. *Hydrological Processes*, *27*(25), 3577–3588. https://doi.org/10.1002/hyp.9486

Li, H., Zhang, Y., Vaze, J., & Wang, B. (2012). Separating effects of vegetation change and climate variability using hydrological modelling and sensitivity-based approaches. *Journal of Hydrology*, *420–421*, 403–418. https://doi.org/10.1016/j.jhydrol.2011.12.033

Li, S., Xu, M., & Sun, B. (2014). Long-term hydrological response to reforestation in a large watershed in southeastern China. *Hydrological Processes*, *28*(22), 5573–5582. https://doi.org/10.1002/hyp.10018

Öztürk, M., Copty, N. K., & Saysel, A. K. (2013). Modeling the impact of land use change on the hydrology of a rural watershed. *Journal of Hydrology*, *497*, 97–109. https://doi.org/10.1016/j.jhydrol.2013.05.022

Peng, H., Tague, C., & Jia, Y. (2016). Evaluating the eco-hydrologic impacts of reforestation in the Loess Plateau , China , using an eco-hydrologic model. *Ecohydrology*, *9*, 498–513. https://doi.org/10.1002/eco.1652

Ristic, R., & Macan, G. (1997). The Impact of erosion control measures on runoff processes. *Human Imparl on Erosion and Sedimentation*, (245), 191–194.

Samraj, P., Sharda, V. N., Chinnamani, S., Lakshmanan, V., & Haldorai, B. (1988). Hydrological behaviour of the Nilgiri sub-w a t ersheds as affected by bluegum plantations, part I. the annual water balance. *Journal of Hydrology*, *103*, 335–345.

Scott, D. F., Prinsloo, F. W., Moses, G., Mehlomakulu, M., & Simmers, A. D. A. (2000). *A re-analysis of the South African catchment afforestation experimental data*.

Silveira, L., & Alonso, J. (2009). Runoff modifications due to the conversion of natural grasslands to forests in a large basin in Uruguay. *Hydrological Processes*, *23*, 320–329. https://doi.org/10.1002/hyp

Silveira, L., Gamazo, P., Alonso, J., & MartÃ-nez, L. (2016). Effects of afforestation on groundwater recharge and water budgets in the western region of Uruguay. *Hydrological Processes*, *30*(20), 3596–3608. https://doi.org/10.1002/hyp.10952

Smith, R., & Scott, D. (1992). The Effects of Afforestation on Low Flows in Various Regions of South-Africa. *Water SA*, *18*(3), 185–194.

Suarez, J. A. R., Perez, R., & Soto, B. (2014). Assessing the influence of afforestation with Eucalyptus globulus on hydrological response from a small catchment in northwestern Spain using the HBV hydrological model. *Hydrological Processes*, *28*, 5561–5572. https://doi.org/10.1002/hyp.10061

Wang, E., Xin, C., Williams, J. R., & Xu, C. (2006). Predicting Soil Erosion for Alternative Land Uses. *Journal of Environment Quality*, *35*(2), 459–467. https://doi.org/10.2134/jeq2005.0063

Zhao, Y., & Yu, X. (2013). Effects of climatic variability and human activity on runoff in the Loess Plateau of China. *The Forestry Chronicle*, *89*(2), 153–161.

Zheng, M., Sun, L., & Yan, M. (2014). Temporal change of runoff and sediment load and their differential response to human activities: A case study for a well-vegetated mountain watershed of southern China. *Journal of Mountain Science*, *11*(1), 73–85. https://doi.org/10.1007/s11629-013-2751-0
